# Exercise suppresses mouse systemic AApoAII amyloidosis through enhancement of the p38 MAPK signaling pathway

**DOI:** 10.1242/dmm.049327

**Published:** 2022-03-21

**Authors:** Xiaoran Cui, Jinko Sawashita, Jian Dai, Chang Liu, Yuichi Igarashi, Masayuki Mori, Hiroki Miyahara, Keiichi Higuchi

**Affiliations:** 1 Department of Aging Biology, Institute of Pathogenesis and Disease Prevention, Shinshu University Graduate School of Medicine, Matsumoto 390-8621, Japan; 2 Products Technology Team, Supplement Strategic Unit, Pharma & Supplemental Nutrition Solutions Vehicle, Kaneka Corporation, Osaka 530-8288, Japan; 3 Department of Neuro-health Innovation, Institute for Biomedical Sciences, Shinshu University, Matsumoto 390-8621, Japan; 4 Aging Biology, Department of Biomedical Engineering, Shinshu University Graduate School of Medicine, Science and Technology, Matsumoto 390-8621, Japan; 5 Community Health Care Research Center, Nagano University Health and Medicine, Nagano 381-2227, Japan

**Keywords:** Amyloidosis, Exercise, Mouse model, Heat shock protein beta-1, P38 MAPK signaling pathway, RNA-seq analysis

## Abstract

Exercise interventions are beneficial for reducing the risk of age-related diseases, including amyloidosis, but the underlying molecular links remain unclear. Here, we investigated the protective role of interval exercise training in a mouse model of age-related systemic apolipoprotein A-II amyloidosis (AApoAII) and identified potential mechanisms. Mice subjected to 16 weeks of exercise showed improved whole-body physiologic functions and exhibited substantial inhibition of amyloidosis, particularly in the liver and spleen. Exercise activated the hepatic p38 mitogen-activated protein kinase (p38 MAPK) signaling pathway and the downstream transcription factor tumor suppressor p53. This activation resulted in elevated expression and phosphorylation of heat shock protein beta-1 (HSPB1), a chaperone that defends against protein aggregation. In amyloidosis-induced mice, the hepatic p38 MAPK-related adaptive responses were additively enhanced by exercise. We observed that with exercise, greater amounts of phosphorylated HSPB1 accumulated at amyloid deposition areas, which we suspect inhibits amyloid fibril formation. Collectively, our findings demonstrate the exercise-activated specific chaperone prevention of amyloidosis, and suggest that exercise may amplify intracellular stress-related protective adaptation pathways against age-associated disorders, such as amyloidosis.

## Abstract (precis)

**Summary:** Interval exercise training delays the progression of mouse systemic amyloidosis, with potential exercise-induced mechanisms that activate the anti-amyloid chaperone HSPB1 enhanced by the p38 MAPK signaling pathway.

## INTRODUCTION

Amyloidosis is a group of diseases characterized by misfolded amyloid precursor proteins accumulating and forming amyloid fibrils that have abundant cross-β conformation. The subsequent extracellular deposition can damage various tissues. Amyloidosis can be divided into systemic and localized amyloidosis. In systemic amyloidosis, amyloid deposits in multiple organs, as seen in familial amyloidotic polyneuropathy (ATTRmt) and senile systemic amyloidosis (ATTRwt) in which amyloid fibrils are formed by mutant or wild-type transthyretin, respectively. In localized amyloidosis, amyloid deposits only in the producing organ, as seen in Alzheimer's disease (AD) and prion disease (Benson et al., 2018). At present, the common etiology of various amyloid diseases is unclear but the development of many types of amyloidosis is likely caused by disrupted protein homeostasis associated with aging (Hipp et al., 2019). Recently, therapies specifically toward reducing the levels of precursor proteins have made progress (Coelho et al., 2013; Song and Yoshizaki et al., 2015), but therapies to reduce existing amyloid deposition have been unsuccessful (Cohen and Wechalekar et al., 2020). Effective treatment strategies should be developed both for delaying amyloidosis onset and its progression. To identify effective treatments, it is essential to understand the molecular mechanisms of amyloid deposition and to use appropriate animal models of amyloidosis.

Apolipoprotein A-II (APOA-II, also known as APOA2) is the second most abundant protein in serum high-density lipoproteins (HDLs). In mice, APOA-II can accumulate to form amyloid fibrils [(apolipoprotein A-II amyloidosis (AApoAII)] associated with age. It deposits extracellularly in various organs but not in the brain (Higuchi et al., 1986). Our previous *in vitro* and *in vivo* studies of mouse AApoAII amyloidosis (Higuchi et al., 1997; Sawashita et al., 2009) showed that AApoAII amyloidosis is induced or transmitted by amyloid fibrils, analogously with mouse models of AD and human prion disease. This occurs through a seeding/nucleation-dependent polymerization event (Jarrett and Lansbury et al., 1993; Xing et al., 2001). Using a mouse AApoAII amyloidosis model system, we recently demonstrated that caloric restriction (Li et al., 2017) and daily supplementation with oxidative stress inhibitors (Dai et al., 2019) effectively slow down progression of amyloidosis. Those data support the concept that preventive therapy is useful for reducing the risk of age-related systemic amyloidosis.

Exercise coordinates complex interconnected systems, achieved through stress hormones and vascular regulation, as well as lipid, insulin and glucose metabolism, thereby improving overall health (Hawley et al., 2014). Regular exercise has been shown to decrease all-cause mortality and cardiovascular risk, as well as the risk of age-related pathologies, including cancer, diabetes mellitus and AD (Wen et al., 2011; Dangardt et al., 2013; Valenzuela et al., 2020). Recent meta-analysis has highlighted that aerobic exercise could be a potential strategy to improve cognitive decline in individuals with AD (Lopez-Ortiz et al., 2021). Indeed, higher levels of habitual exercise are related to lower levels of brain amyloid beta (Aβ) burden in AD patients (Brown et al., 2017). A study using a mouse model of AD demonstrated that aerobic wheel running reduces cerebral Aβ deposition and improves spatial memory (Garcia-Mesa et al., 2016). However, the understanding of molecular links between exercise intervention and disease prevention is still lacking.

An interval walking training system (IWT) developed at Shinshu University School of Medicine is effective for increasing physical fitness and decreasing scores for lifestyle-related diseases (Nemoto et al., 2007; Masuki et al., 2019; Masuki et al., 2020). IWT is one such regimen in which the individual engages in alternating fast and slow walking for 3-min intervals that are equivalent to >70% and ∼40% of individual peak aerobic capacity (VO_2peak_), respectively. These repetitions of muscle contraction and relaxation at the required intensity, like traditional resistance exercise, lead to increases in thigh muscle mass and strength, and VO_2peak_ in older humans, suggesting that IWT combines resistance and aerobic training (Nemoto et al., 2007). In this study, we developed an interval training (IT) protocol for mice that mimics the human IWT and used it in a unique mouse model of age-related systemic AApoAII amyloidosis. Our current data show that exercise effectively delays the progression of systemic amyloidosis, especially in the liver and spleen. Further mechanistic analyses suggested that the stress-sensitive p38 mitogen-activated protein kinase (p38 MAPK) signaling pathway upregulated by exercise is further activated by the unfolded protein response with amyloid deposition, leading to elevated expression and phosphorylation of the molecular chaperone HSPB1.

## RESULTS

### IT improves physiological characteristics

**Fig. 1.**
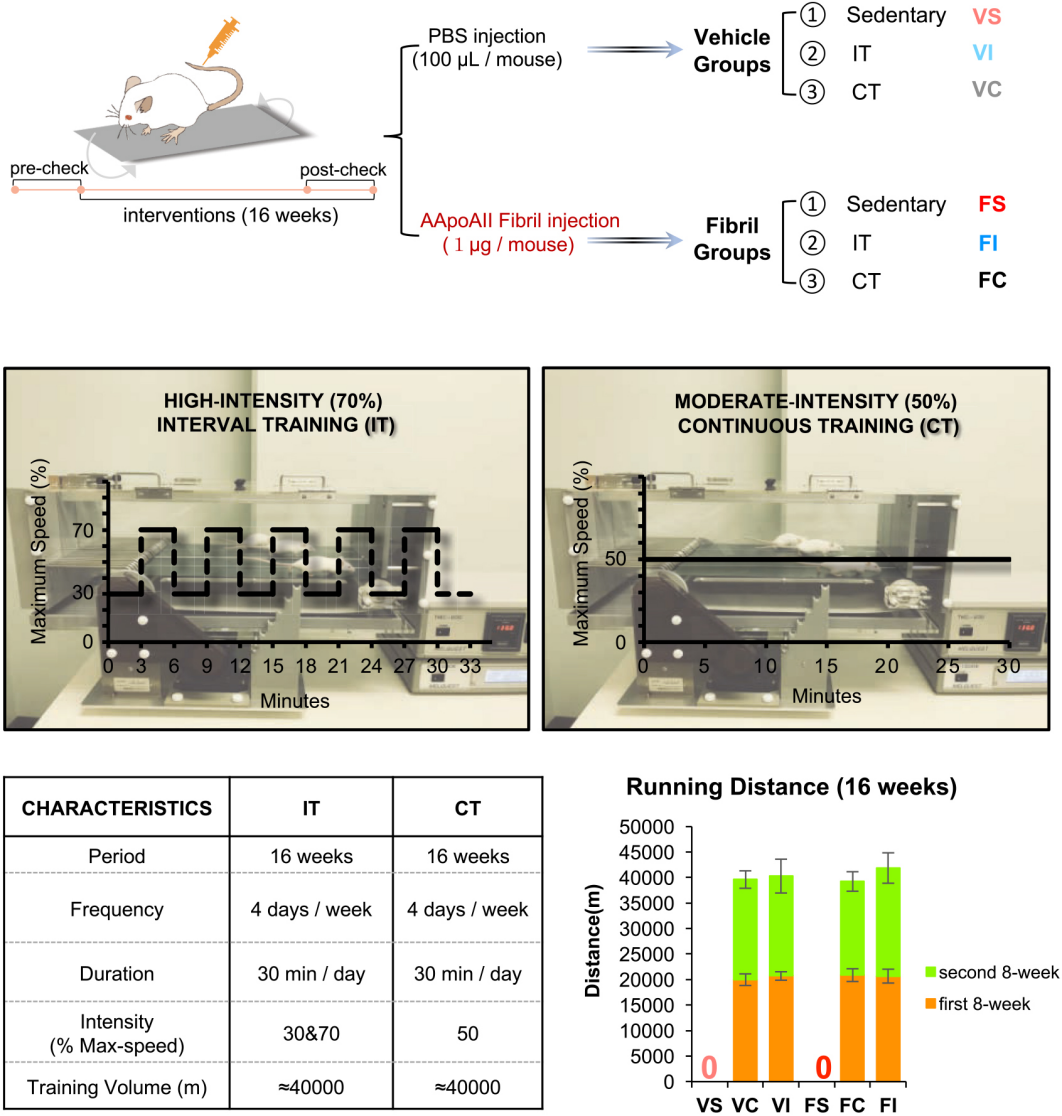
**Experimental Design.** The detailed experimental design is described in the Materials and Methods. Eight-week-old female R1.P1-*Apoa2^c^* mice were randomly divided into six groups. Three groups, VS (sedentary), VI (high-intensity interval exercise, IT) and VC (moderate-intensity continuous exercise, CT) had no amyloidosis induction and were injected with saline vehicle. The other three groups, FS (sedentary), FI (IT) and FC (CT) had amyloidosis induction by injection of AApoAII fibril via tail veins. Amyloidosis was induced at the same time that the exercise protocols were initiated. Before these interventions, all mice were first acclimated to the treadmill. Then, the maximal running speed (V_max_) test was performed, and various physiological indices were measured (pre-check). Subsequently, each mouse was subjected to 16 weeks of either (1) IT (repeated cycles of 3 min at 70% V_max_ and 3 min at 30% V_max_ for 30 min per day, 4 days/week) or (2) CT (30 min at 50% V_max_ per day, 4 days/week) on a treadmill. Except for the exercise type and intensity, the period, frequency, duration and training volume were the same between IT and CT. Bars show the running distance after 16 weeks of exercise; error bars represent the mean±s.d. Two weeks before the end of these interventions (post-check), the V_max_ test and physiological indices were measured again. The serum samples and organs of mice were collected within 24 h following training.

**Fig. 2.**
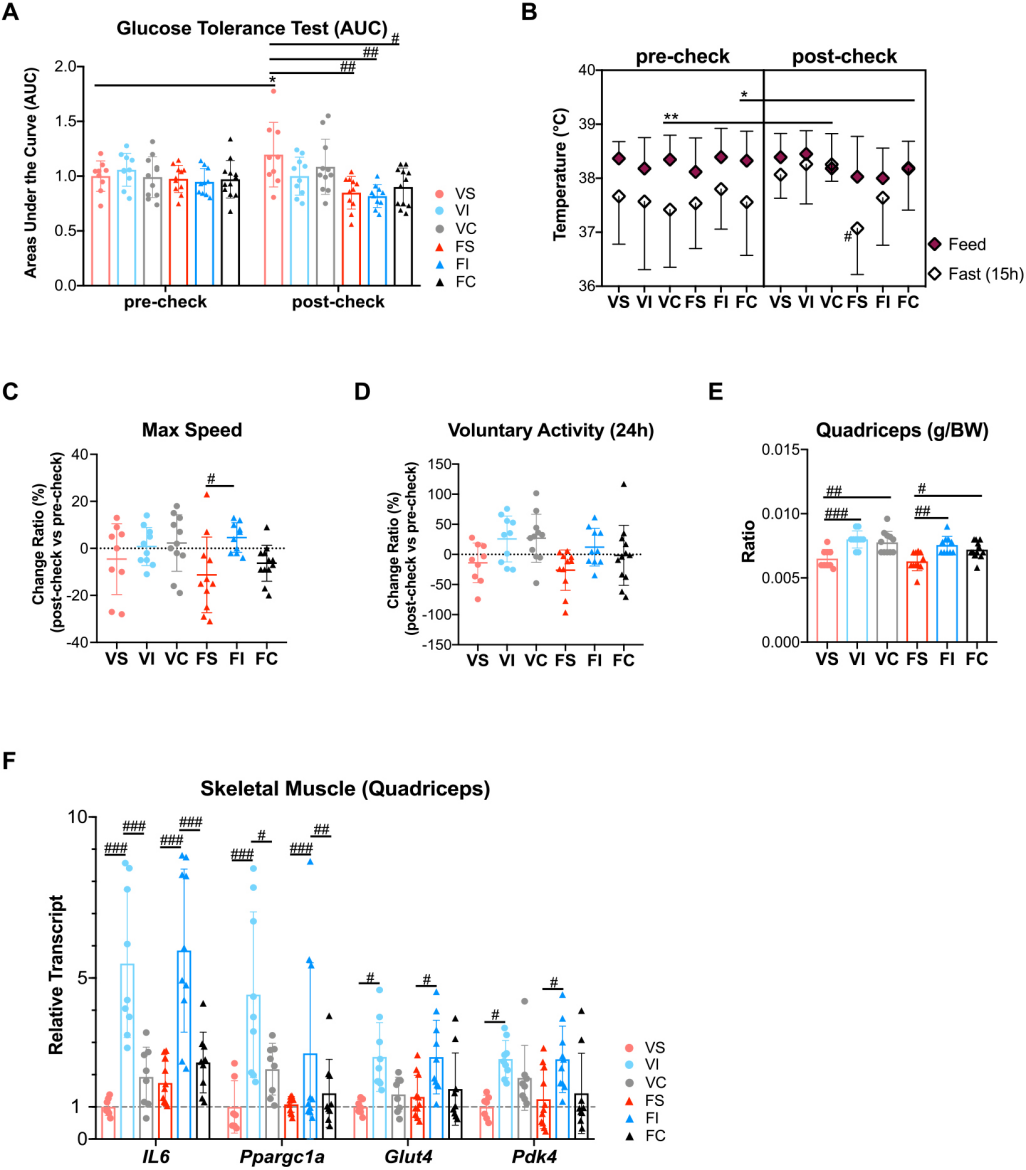
**Comparative analysis of the physiological benefits of exercise training.** (A) Exercise improved age-related glucose intolerance. An IGTT was performed in the pre-check and post-check, and AUC were calculated. (B) Exercise maintained constant body temperature after a 15-h fast, especially in the amyloidosis-induced groups. The feeding and 15-h fasting body temperatures are shown. (C,D) The change ratios of maximal running speed (C) and 24-h voluntary activity (D) tended to be elevated after training. (E) The two types of exercise increased the ratio of quadriceps weight to body weight after training. (F) IT had a persistent effect on the mRNA levels of muscle genes. Real-time qPCR was used to assess the mRNA expression of genes related to energy metabolism in quadriceps from mice after training. Data were normalized to 18S. Each dot represents an individual mouse (A,C-F) (VS, *n=*9; VI, *n*=10; VC, *n*=11; FS, *n=*11; FI, *n*=10; FC, *n=*12). Data are mean±s.d. *P<0.05, **P<0.01 versus respective pre-check (Repeated-measures ANOVA). ^#^*P*<0.05, ^##^*P*<0.01; ^###^*P*<0.001 (one-way ANOVA with Tukey–Kramer test for comparison between groups).

To investigate the effects of exercise on physical function and AApoAII amyloidosis, female R1.P1-*Apoa2^c^* mice underwent IT or training volume-matched moderate-intensity continuous exercise training (CT) for 16 weeks (Fig. 1). The body weights of mice among groups were not different across the 16-week training period (Fig. S1, Fig. S2A). After training, the weight of white adipose tissue in IT groups tended to be reduced compared to sedentary mice, but serum lipid profiles (triglyceride, total cholesterol and HDL cholesterol) were not different among groups (Fig. S2B,C). Blood pressure increased with age in sedentary mice but it improved after training (Fig. S2D). Intraperitoneal glucose tolerance tests (IGTTs) and the areas under the curve (AUC) showed that in vehicle groups, age-related deterioration of glucose tolerance was observed for sedentary mice (VS), but both IT exercise (VI) and CT exercise (VC) prevented this deterioration (Fig. S2E; Fig. 2A). Additionally, the AUC in amyloidosis-induced groups regardless of exercises was lower compared with vehicle groups in the post-check (Fig. S2E; Fig. 2A). The 15-h fasting temperature decreased with age in amyloidosis-induced sedentary mice (FS) but exercises rescued this decline and reduced the difference between feeding and fasting temperatures in amyloidosis-induced IT mice (FI) and amyloidosis-induced CT mice (FC), indicating that exercise better maintained constant temperature after fasting (Fig. 2B). Maximal running speed (V_max_) and 24-h voluntary activities tended to increase in exercised groups, particularly in amyloidosis-induced IT mice (FI; Fig. 2C,D). Of note, both IT and CT mice exhibited higher quadriceps muscle mass relative to sedentary mice after training (Fig. 2E).

Muscle is the organ most directly affected by exercise. Thus, we determined the molecular responses. Interleukin-6 (IL6) is a myokine involved in muscle-liver and muscle-systemic crosstalk, and it has a role in glucose uptake in muscle cells (Pedersen and Febbraio et al., 2012). *Il6* mRNA levels in quadriceps were dramatically upregulated more than 5-fold by IT compared to sedentary mice, regardless of influence by AApoAII amyloidosis, whereas *IL6* mRNA levels in the CT groups were elevated ∼2-fold (Fig. 2F). Quadriceps from the IT mice had higher mRNA levels of the mitochondria regulator gene peroxisome-proliferator-activated receptor γ coactivator 1α (*Ppargc1a*), the glucose uptake regulating gene glucose transporter 4 (*Glut4*, also known as *Slc2a4*) and the fatty acid oxidation biomarker gene pyruvate dehydrogenase kinase 4 (*Pdk4*) (Fig. 2F). Together, these results indicate a healthier physiological profile in exercised mice compared with sedentary mice.

### IT and CT suppress AApoAII amyloid deposition

**Fig. 3.**
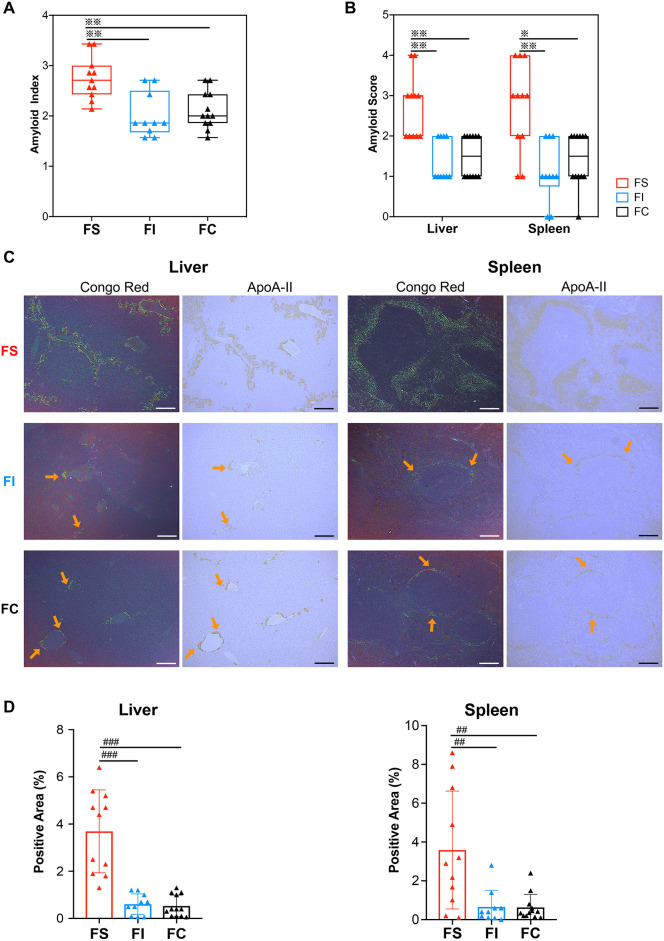
**IT and CT suppress AApoAII amyloid deposition in the liver and spleen.** (A) Amyloid index of amyloidosis-induced groups. (B) Amyloid scores in the liver and spleen from mice in amyloidosis-induced groups. (C) Representative light microscope and IHC images of AApoAII deposition in the livers and spleens from mice in amyloidosis-induced groups. Amyloid deposits (indicated by orange arrows) were identified by apple-green birefringence of Congo Red-stained tissue sections under a polarized light microscope (left panels). AApoAII deposition was confirmed as a brown color seen with IHC with anti-APOA-II antiserum (right panels). Scale bars: 100 μm. (D) Quantification of positive areas of amyloid deposition in the livers and spleens seen with IHC with anti-APOA-II antiserum. Each dot represents an individual mouse (A,B,D) (FS, *n=*11; FI, *n*=10; FC, *n=*12). Box and whisker plots in A and B represent, respectively, the amyloid index (i.e. the average amyloid scores for the seven organs examined) and the amyloid score (i.e. the degree of amyloid deposition in liver and spleen). The boxes represent the 25th, median (black line), mean (cross) and 75th percentiles of the data; the whiskers represent the lowest (or highest) data within 1.5 interquartile range from the 25th (or 75th) percentile range. ^※^*P<*0.05, ^※※^*P*<0.01, versus FS (Kruskal–Wallis test with the Steel–Dwass test). Positive areas of AApoAII amyloid are shown as mean±s.d. (D). ^##^*P*<0.01; ^###^*P*<0.001 (one-way ANOVA with the Tukey–Kramer test for comparison between groups).

To further investigate the effects of exercise on AApoAII amyloidosis, mouse organs were obtained within 24 h of completing the 16-week training. AApoAII amyloid deposition was analyzed by apple-green birefringence in Congo Red-stained tissue sections and immunohistochemical staining (IHC) with anti-APOA-II antiserum. Vehicle groups without amyloidosis induction showed no amyloid deposition (data not shown). The amyloid index, a semi-quantitative parameter for evaluating the degree of systemic AApoAII deposition, was significantly lower in both FI and FC groups compared with the FS group (Fig. 3A). Livers and spleens from mice in the amyloidosis-induced groups that underwent IT and CT showed significant and similar alleviations of AApoAII amyloid deposition (Fig. 3B,C). To quantify amyloid deposition in the liver and spleen, the ratios of areas positively stained with anti-APOA-II antiserum to the whole section area were calculated. This quantification confirmed that AApoAII amyloid deposition was indeed dramatically suppressed in the liver and spleen following both exercise regimens (Fig. 3D). Additionally, amyloid deposition in the other examined organs tended to decrease in exercised mice, particularly in the FI group, but this difference did not attain significance (Fig. S3). These results suggest that both exercise regimens induce organ-dependent suppression of amyloidosis.

The level of amyloid precursor protein is an important risk factor that has a positive correlation with the amyloid deposition in almost all amyloidoses (Coelho et al., 2013; Song and Yoshizaki et al., 2015; Wechalekar et al., 2016). Apolipoprotein A-I (APOA-I) and precursor protein APOA-II are mainly found in serum HDL, and account for ∼75% and ∼20%, respectively, of apolipoproteins in HDL. Exercises did not affect either the serum levels of APOA-II in vehicle groups or the APOA-II mRNA levels in liver (Fig. S4). We observed lower APOA-II serum levels and higher APOA-I serum levels, as well as lower APOA-II/APOA-I ratios in the amyloidosis-induced groups (Fig. S4), indicating that serum APOA-II deposited into AApoAII amyloid fibrils.

### Transcriptome analysis reveals a significant increase of p38 MAPK and Hspb1 in response to IT and amyloidosis

**Fig. 4.**
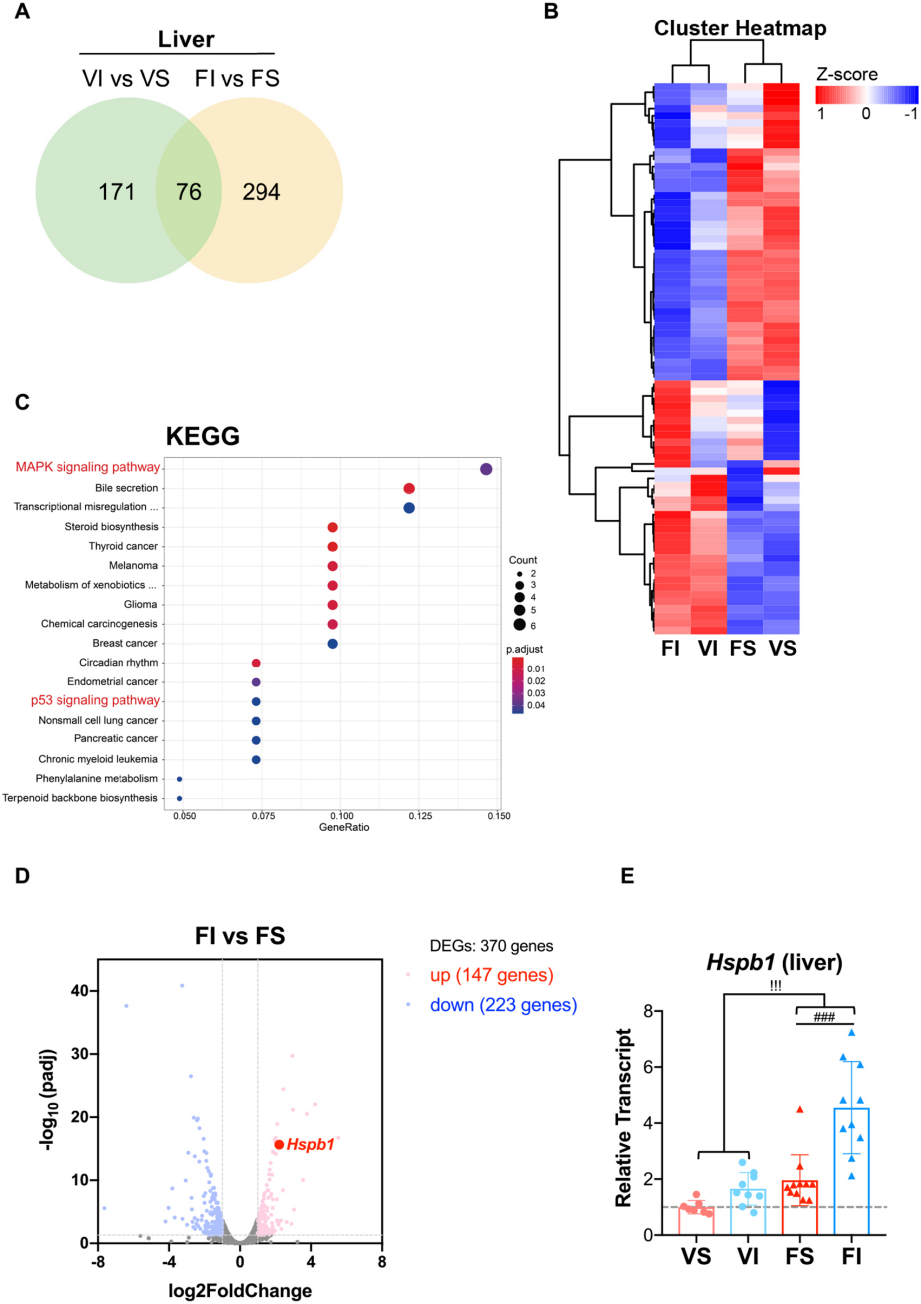
**Transcriptome characteristics of amyloidosis-alleviated livers in IT mice.** (A) Venn diagram showing DEGs in the vehicle and fibril groups. Genes displayed significant differential expression based on a log_2_-fold change >1 and corrected *P*<0.05. (B) Heatmap of cluster analysis of the overlap of 76 DEGs showing that they were only associated within the response to IT. (C) For analysis of IT-mediated alleviation of amyloidosis, the most significantly enriched KEGG pathways of the overlap of 76 DEGs are shown. The greatest number of DEGs was implicated in the MAPK signaling pathway. (D) Volcano plot diagram showing upregulated and downregulated DEGs in FI versus FS. P38 MAPK pathway-related gene *Hspb1* as a chaperonin was significantly upregulated by IT (log_2_-fold change >2.2 and corrected *P*<0.000). (E) Real-time qPCR of *Hspb1* mRNA normalized to 18S in the liver of mice after 16-week interventions. Each dot represents an individual mouse (VS, *n=*7; VI, *n*=9; FS, *n=*11; FI, *n*=10). Data are mean±sd. ^###^*P*<0.001 (one-way ANOVA with Tukey–Kramer method for comparison between groups). ^!!!^*P*<0.001 (two-way ANOVA for comparison of the magnitude of changes between different groups in mice with or without amyloidosis induction).

To identify the potential signaling pathways or effector molecules for IT-mediated prevention of amyloidosis, we performed RNA-seq analysis to investigate transcriptome changes in response to IT and amyloidosis in the liver. Analysis of differentially expressed genes (DEGs; >2-fold change and corrected *P*<0.05) showed that IT induced 247 DEGs in vehicle groups (VI versus VS) and 370 DEGs in fibril groups (FI versus FS) (Fig. 4A; Table S2). The overlapping 76 DEGs between the vehicle groups and fibril groups showed common profiles in response to IT regardless of amyloidosis (Fig. 4B; Table S2). Enrichment analysis of the 76 genes based on the Kyoto Encyclopedia of Genes and Genomes (KEGG) pathway database identified several pathways, including MAPK and p53 signaling pathways that significantly responded to IT. The highest number of DEGs was implicated in the MAPK signaling pathway (Fig. 4C). Among the genes involved in this signaling pathway, p38 MAPK axis-related gene heat shock protein beta-1 (*Hspb1*) was significantly upregulated over 4.5-fold in response to IT in amyloidosis-induced mice (Fig. 4D). Consistent with this finding, the mRNA levels of *Hspb1* were upregulated ∼2-fold either in response to IT (VI) or amyloid deposition (FS), and were additively enhanced by IT in the presence of amyloidosis (FI) compared with the VS group (Fig. 4E). Here, we concentrated on *Hspb1* because it encodes for a small heat shock protein that functions as a molecular chaperone binding to misfolded polypeptides to reduce abnormal protein aggregation (Benarroch, 2011). An earlier study suggested that it has a neuroprotective effect in AD and Parkinson's disease (Muchowski and Wacker et al., 2005).

### IT-dependent increase in p-HSPB1 might play a protective role against AApoAII amyloidosis

**Fig. 5.**
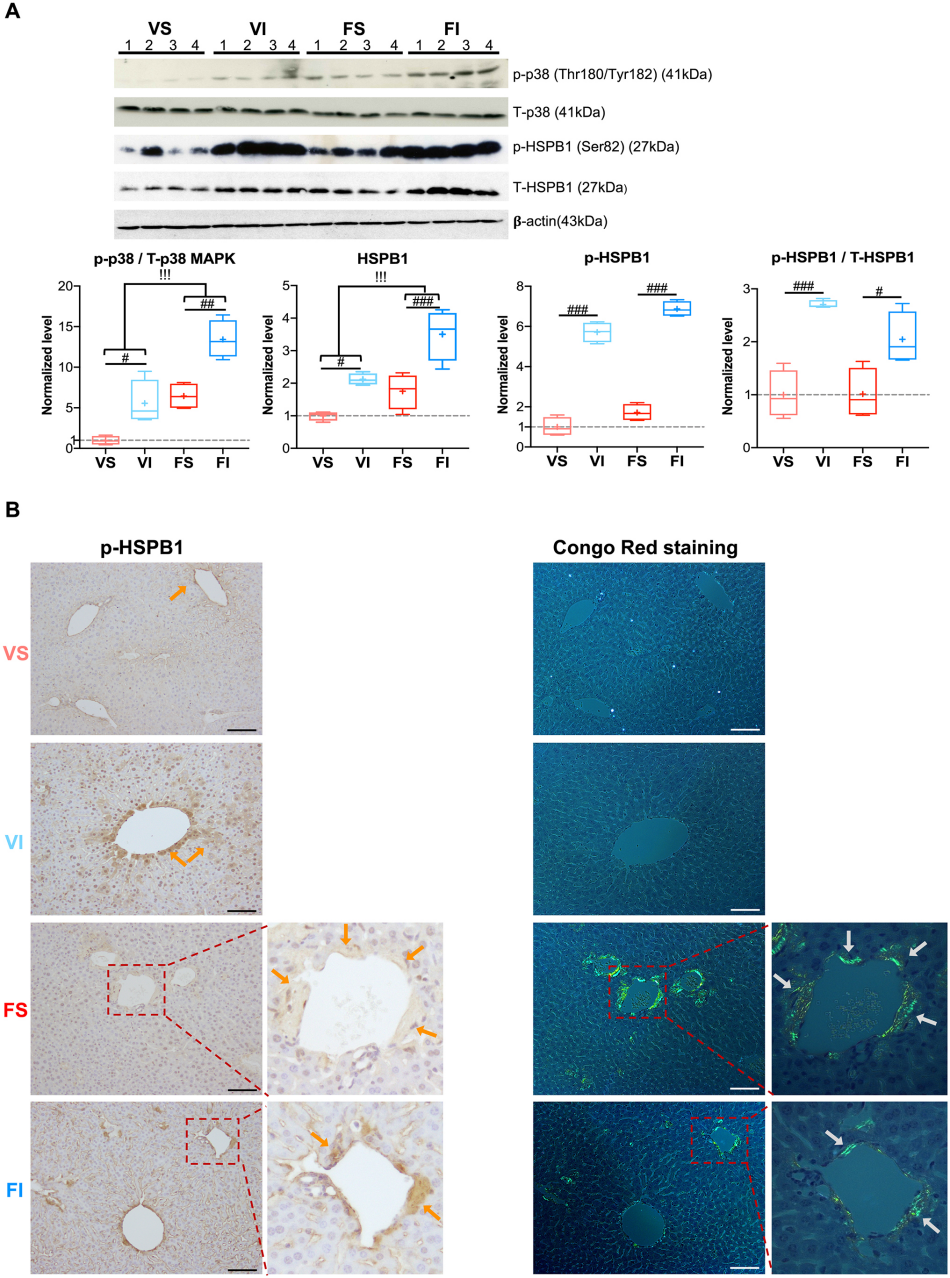
**IT-dependent induced p-HSPB1 might play a protective role against AApoAII amyloidosis.** (A) Western blot analysis and quantification of phosphorylated p38 MAPK (Thr180/Tyr182), total p38 MAPK, p-HSPB1 (Ser82) and total HSPB1 protein in the livers of mice after 16-week interventions. Box and whisker plots show fold changes relative to the VS group. The boxes represent the 25th, median (black line), mean (cross) and 75th percentiles of the data; the whiskers represent the lowest (or highest) data within 1.5 interquartile range from the 25th (or 75th) percentile range. (B) Representative images of IHC for p-HSPB1 and Congo Red birefringence of amyloid deposition in the livers of mice after 16-week interventions. p-HSPB1 and amyloid deposition are indicated by orange and gray arrows, respectively. Scale bars: 50 μm. Data are mean±s.d. (VS, *n*=7; VI, *n*=8; FS, *n*=8; FI, *n*=8). ^#^*P*<0.05, ^##^*P*<0.01, ^###^*P<*0.001 (one-way ANOVA with Tukey–Kramer method for comparison between groups). ^!!!^*P*<0.001 (two-way ANOVA for comparison of the magnitude of changes between different groups in mice with or without amyloidosis induction).

We next inquired whether IT contributed to elevated phosphorylation of HSPB1 (p-HSPB1), as it is known that the p38 MAPK signaling cascade phosphorylates HSPB1 under conditions of stress (Zarubin and Han et al., 2005). Recent studies have demonstrated that stress-induced phosphorylation of HSPB1 enhances its chaperone activity against amyloid fibril formation *in vitro* (Jovcevski et al., 2015; Liu et al., 2020). Western blot analysis showed significantly higher phosphorylated p38 MAPK and total HSPB1 protein levels in the IT groups (Fig. 5A). Notably, levels of both phosphorylated p38 MAPK and total HSPB1 were additively enhanced in amyloidosis-induced IT mice (FI) compared to the VS group (Fig. 5A). Thus, the levels of total HSPB1 protein were coordinately upregulated with the change in phosphorylated p38 MAPK (Fig. 5A). In contrast, higher phosphorylation of HSPB1 only occurred with IT.

We observed accumulation of p-HSPB1 in amyloid deposition areas using IHC with p-HSPB1 antibody and Congo Red staining of paraffin-embedded liver sections (Fig. 5B). IT induced greater amounts of p-HSPB1, indicated by a brown stain that reacted with antibody against p-HSPB1 in hepatocytes cytoplasm and at amyloid deposition sites (Fig. 5B). This observation was in line with the western blot analysis. Colocalization of p-HSPB1 and extracellular AApoAII amyloid deposition may suggest an interaction between p-HSPB1 and extracellular AApoAII amyloid deposition.

We subsequently investigated whether Hspb1 played a protective role against amyloid deposition in the spleen (Fig. 3B) and lungs (Fig. S5A), where IT reduced (spleen) or did not reduce (lungs) amyloid deposition. Real-time qPCR of *Hspb1* mRNA expression and western blot analysis of total and phosphorylated HSPB1 levels in the spleen showed similar results as that for the liver (Fig. S5B). Similarly, we observed large amounts of p-HSPB1 accumulated at amyloid deposition sites in the FI group in the spleen (Fig. S5C). On the other hand, mRNA expression and total protein levels of HSBP1 remained unchanged by either IT or amyloid deposition in the lungs (Fig. S5D). Therefore, we consider the induction of p-HSPB1 to be one possible mechanism for IT-mediated prevention of AApoAII amyloidosis.

### The p53 signaling pathway upregulates Hspb1 levels in mice with IT

**Fig. 6.**
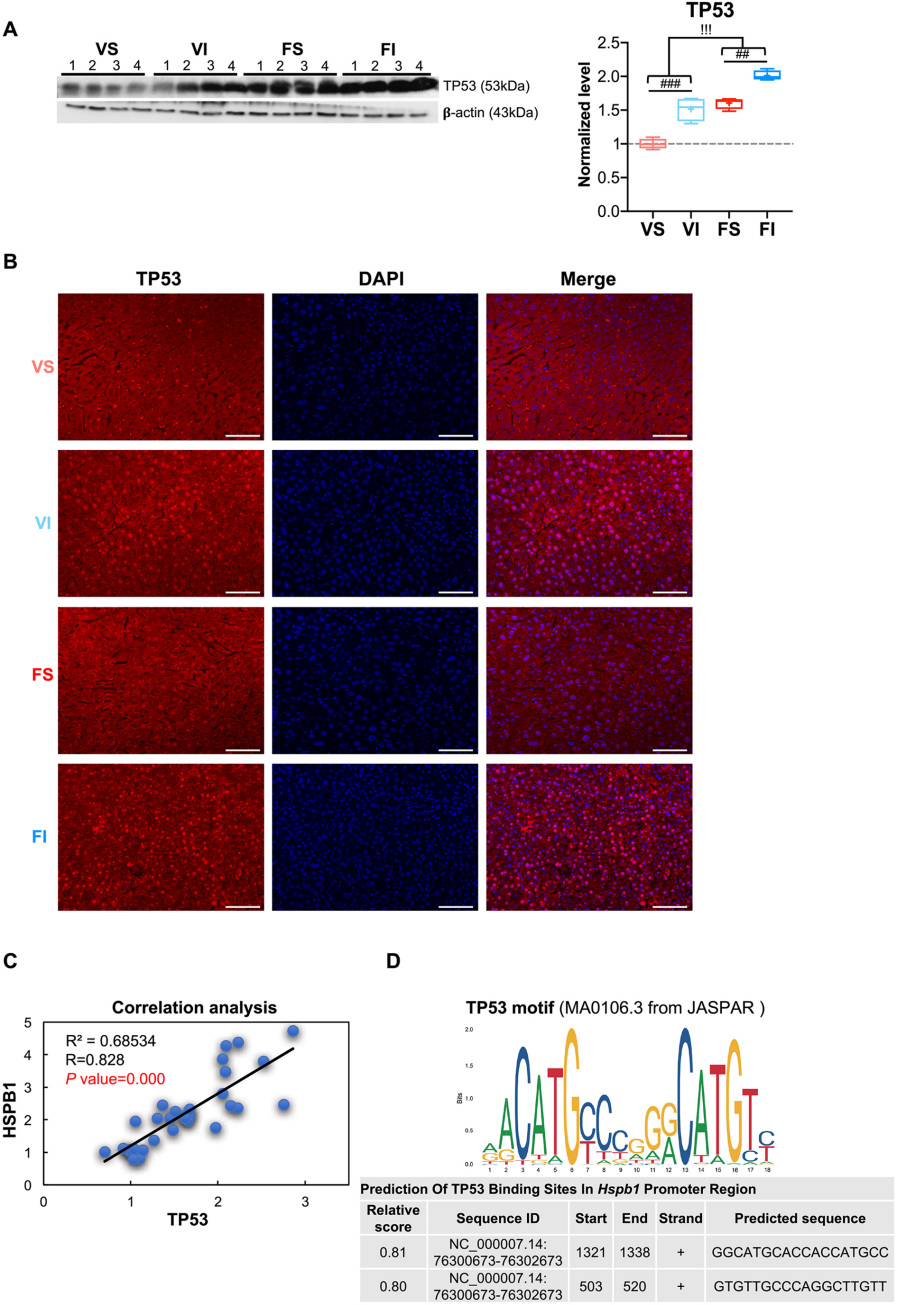
**p53 signaling pathway upregulated Hspb1 levels in mice with IT.** (A) Western blot analysis and quantification of TP53 protein in the livers of mice after 16-week interventions. Box and whisker plots show fold changes relative to the VS group. The boxes represent the 25th, median (black line), mean (cross) and 75th percentiles of the data; the whiskers represent the lowest (or highest) data within 1.5 interquartile range from the 25th (or 75th) percentile range. (B) Immunofluorescence determination of TP53 in the liver after 16-week interventions. Scale bars: 50 μm. (C) Two-tailed Pearson's correlation analysis between TP53 and HSPB1 proteins in the livers of mice after 16-week interventions (31 female mice). (D) Predictive analyses coupled with database-deposited data (JASPAR open-access database) suggested that TP53 binds the promoter of the *Hspb1* gene. Data are mean±s.d. (VS, *n=*7; VI, *n*=8; FS, *n=*8; FI, *n*=8). ^##^*P*<0.01; ^###^*P*<0.001 (one-way ANOVA with Tukey–Kramer method for comparison between groups). ^!!!^*P<*0.001 (two-way ANOVA for comparison of the magnitude of changes between different groups in mice with or without amyloidosis induction).

The tumor suppressor protein p53 is a well-defined downstream transcription factor of p38 MAPK under stress conditions, such as physical exercise (Hoene and Weigert et al., 2010; Bartlett et al., 2014). Western blot analysis of liver lysates from vehicle and fibril groups showed that p53 protein (TP53) levels were elevated by IT relative to sedentary mice (Fig. 6A). Mice with amyloidosis also exhibited higher TP53 levels compared with vehicle groups. Importantly, IT and amyloid deposition (FI) additively enhanced TP53 levels (Fig. 6A), data consistent with the change of phosphorylated p38 MAPK and total HSPB1 protein levels (Fig. 5A). Immunofluorescence analysis of TP53 revealed that IT significantly enhanced intranuclear signal intensities both in vehicle and fibril groups (Fig. 6B). Intranuclear fluorescence signals for TP53 were also observed in the FS group compared with the VS group (Fig. 6B). Growth arrest and DNA-damage-inducible 45 gamma (*Gadd45g*) and cyclin-dependent kinase inhibitor 1A (*Cdkn1a*) are IT-induced DEGs involved in the p53 signaling pathway and transcriptionally regulated by p53 (Levine, 2020). Both of their mRNA levels were significantly upregulated either by IT (VI) or amyloid deposition (FS), and were additively enhanced by IT in the presence of amyloidosis (FI) compared with the VS group (Fig. S6A). PGC-1α, which is an exercise-related downstream substrate of p38 MAPK (Hawley et al., 2014), was upregulated in response to IT but not by amyloid deposition (Fig. S6B).

The levels of mRNA for the common molecular chaperones heat shock protein family A (HSP70) Member 1B (*Hspa1b*) and crystallin alpha B (*Cryab*), which are primarily regulated by the transcription factor heat shock factor 1 (HSF1) (Benarroch, 2011; Kourtis and Tavernarakis et al., 2011), showed no change in response to either IT or amyloidosis in the liver (Fig. S6C). Therefore, IT-induced higher expression of *Hspb1* in AApoAII amyloidosis might be independent of HSF1 and regulated by other transcription factors.

We found that the levels of HSPB1 and TP53 proteins induced by IT and amyloidosis were similar (Fig. 5A, Fig. 6A). Pearson analysis of those protein levels in the liver showed a strong positive correlation (*P*<0.001, Fig. 6C). Predictive analyses of the putative binding site for TP53 in the transcriptional regulatory region of *Hspb1* revealed a homology higher than 80% with the 18-mer TP53-motif sequence. That finding suggested that the *Hspb1* gene may be regulated by TP53 (Fig. 6D). These data suggest that IT-mediated activation of p38 MAPK might induce TP53-dependent transcriptional regulation of *Hspb1*.

### Amyloid deposition induces the unfolded protein response and activated p38 MAPK

**Fig. 7.**
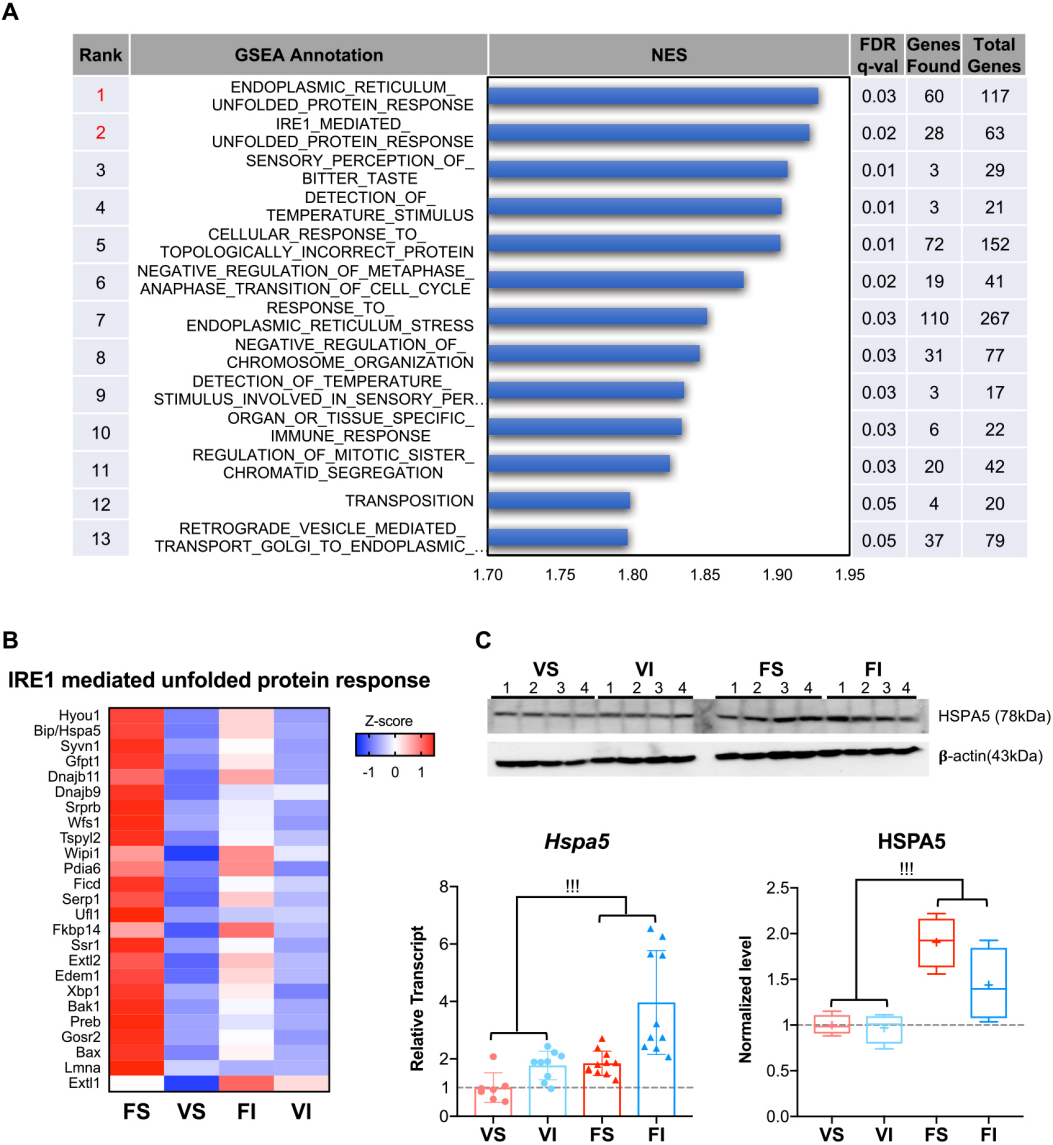
**Transcriptional changes in the livers of amyloidosis-induced sedentary mice.** (A) GSEA based on GO showed UPR-related terms were significantly over-represented in sedentary mice with amyloidosis. FDR, false discovery rate; NES, normalized enrichment scores. (B) Heatmap analysis of the gene set in IRE1-mediated UPR identified by GSEA. (C) Real-time qPCR (VS, *n=*7; VI, *n*=9; FS, *n=*11; FI, *n*=10) and western blot (*n=*4) validations of UPR primary sensor HSPA5 in the livers of mice after 16-week interventions. Bars show the quantification of real-time qPCR, box and whisker plots show the results of western blots. Both graphs show fold changes relative to the VS group. The boxes represent the 25th, median (black line), mean (cross) and 75th percentiles of the data; the whiskers represent the lowest (or highest) data within 1.5 interquartile range from the 25th (or 75th) percentile range. Each dot represents an individual mouse. Data are mean±s.d. ^!!!^*P*<0.001 (two-way ANOVA for comparison of the magnitude of changes between different groups in mice with or without amyloidosis induction).

We sought to identify gene clusters that were related to amyloid deposition and were also associated with the activation of p38 MAPK by amyloid deposition. Thus, we performed gene set enrichment analysis (GSEA) in mice with and without amyloidosis based on RNA-seq. GSEA categorized by gene ontology (GO) analysis illustrated that (1) the endoplasmic reticulum (ER) unfolded protein response (UPR) and (2) inositol-requiring 1 transmembrane kinase endonuclease 1 (IRE1, also known as ERN1)-mediated UPR were among the highest ranked terms (Fig. 7A). A heatmap of the expression of 25 genes related to the IRE1-mediated UPR showed higher expression in sedentary mice with amyloidosis (FS) compared to vehicle sedentary mice (VS) (Fig. 7B). IT alleviated amyloidosis and IRE1-mediated UPR in amyloidosis-induced mice (Fig. 7B). We validated mRNA and protein levels of binding immunoglobulin protein (HSPA5, also known as BIP), which is a major IRE1-mediated UPR protein and serves as a primary sensor in the activation of the UPR (Walter and Ron et al., 2011). Both mRNA expression and protein levels of HSPA5 were higher in amyloidosis-induced mice compared with vehicle groups (Fig. 7C). IRE1 has been linked to activation of MAPK, especially p38 MAPK (Matsuzawa et al., 2002). Additionally, the heatmap analysis of genes involved in ER-UPR showed similar results as that for IRE1-mediated UPR (Fig. S7).

## DISCUSSION

**Fig. 8.**
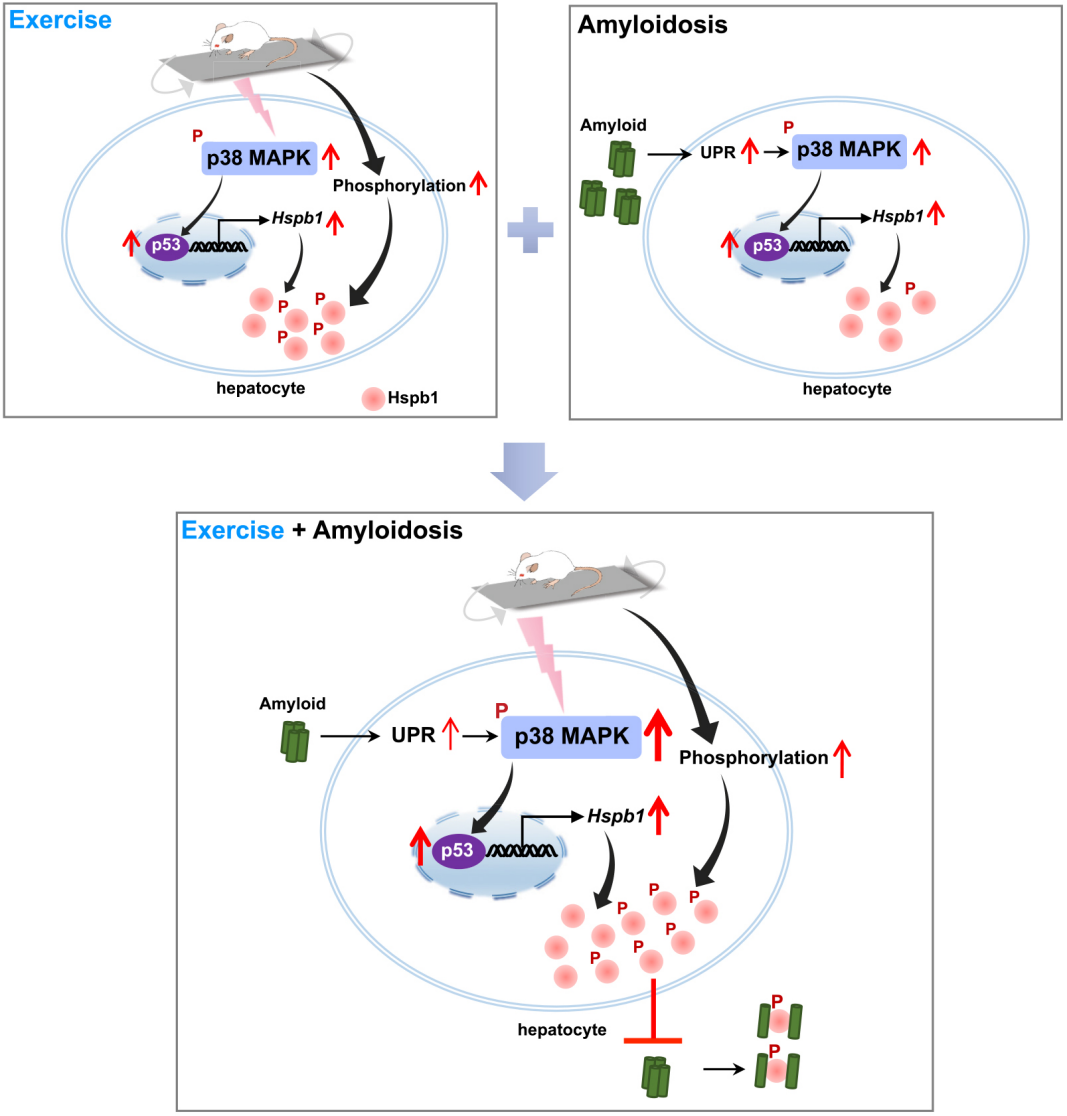
**A potential molecular mechanism underlying IT exhibits an anti-amyloid effect.** Here, we propose a novel molecular mechanism in which the p38 MAPK-p53-HSPB1 axis could play a key role in the anti-amyloid load. The p38 MAPK-p53-HSPB1 axis showed upregulation in the livers of mice with exercise (top left) or systemic AApoAII amyloidosis alone (top right). In particular, phosphorylated HSPB1 showed an exercise-dependent increase. Exercise additively enhanced this phenomenon in the amyloid-reduced liver and greatly increased levels of phosphorylated HSPB1 outside the cell. Phosphorylated HSPB1 colocalized with amyloid depositions, which in turn could inhibit fibril formation (bottom).

Understanding the biological mechanisms underlying improved fitness could lead to valuable treatments that reduce the risk of age-related pathogenesis and could simultaneously reveal new pharmacological targets. At present, therapies against amyloidosis, including caloric restriction, small interfering RNA and antibody-based drugs, are focused on the reduction of precursor proteins (Coelho et al., 2013; Song and Yoshizaki et al., 2015; Li et al., 2017). In this study, our evidence revealed that a relatively long-term (16 weeks) IT regimen significantly halted disease progression of systemic amyloidosis without affecting the levels of precursor protein APOA-II. Transcriptome assays in the liver followed by validation analyses led to the following conclusions: (1) IT markedly activated the hepatic p38 MAPK signaling pathway, resulting in the activation of TP53, and upregulated the expression and phosphorylation of HSPB1; (2) AApoAII amyloid deposition induced UPR, activating the hepatic p38 MAPK signaling pathway, leading to higher expression of HSPB1 but not phosphorylated HSPB1; (3) AApoAII amyloid deposition additively enhanced the IT-activated p38 MAPK signaling pathway; and (4) IT induced the greater amounts of phosphorylated HSPB1 at sites of AApoAII amyloid deposition in the liver and spleen. As HSPB1 is a unique molecular chaperone identified from our transcriptome sequencing, we propose a potential mechanism whereby the upregulation of the p38 MAPK signaling pathway could be a key event for exercise adaptation and the prevention of amyloidosis (Fig. 8).

P38 MAPK is an essential transduction mediator involved in gene expression and metabolic regulation in response to various extracellular stimuli (de Nadal et al., 2011; Lawan and Bennett et al., 2017). Those stimuli range from environmental to intracellular pressures, such as exercise, oxidative stress (reactive oxygen species), DNA damage and inflammation (Kramer and Goodyear et al., 2007; Canovas and Nebreda et al., 2021). Multiple lines of evidence have suggested that aerobic exercise in rodents, as well as cycling and marathon running in humans, can potently increase the phosphorylation of p38 MAPK in skeletal muscle or liver (Nader and Esser et al., 2001; Kramer and Goodyear et al., 2007; Hoene et al., 2010). Here, we observed an approximate 6-fold increase in phosphorylated p38 MAPK levels in the liver with exercise (Fig. 5). Notably, in amyloidosis-induced mice, the levels of phosphorylated p38 MAPK were further doubled by exercise. This result suggests that exercise could enhance the p38 MAPK-dependent protective adaptation pathway against harmful stress. Moreover, we found a higher mRNA expression level of glucose transporter *Glut4* in the training quadriceps. The increased glucose uptake by working muscles can stimulate hepatic glucose output into the circulation to maintain blood glucose levels. We observed mRNA levels of gluconeogenesis regulation-related DEGs, including *Ppargc1a*, *Pdk4* and glucose-6-phosphatase (*G6pc*), were upregulated by exercise in the liver (Fig. S8), indicating a decline in plasma glucose concentrations. Weigert et al. showed that the lower plasma glucose levels after running are related to the activation of p38 MAPK signaling protein in the liver (Hoene et al., 2010).

We found that exercise upregulated mRNA levels of *Gadd45g and Cdkn1a*, both of which are transcriptionally regulated by p53. P38 MAPK activates p53 through direct phosphorylation, and it subsequently regulates the transcription of downstream genes (de Nadal et al., 2011). The activation of p53 increases its stability and leads to accumulation in the nucleus (Inoue et al., 2005; Marine, 2010), which is consistent with our data (Fig. 6). As a stress-responsive protein, p53 has a well-documented role in protecting against cancer development. Moreover, it is now becoming clear that p53 can contribute to mitochondrial biogenesis, life expectancy and overall fitness of an organism (Bartlett et al., 2014; Levine, 2020). Based on current evidence, p53 can play two important but fundamentally opposing roles in coping with stress, namely, inducing either cell survival or cell death according to the activation level of stress (Vousden and Lane et al., 2007). Of note, in this study we observed that the number of apoptotic cells was not affected by the degree of p53 activation (Fig. S9) but instead upregulated HSPB1 expression. It has been reported that wild-type p53 induces HSPB1 expression *in vitro* (Gao et al., 2000). In line with this, we propose that the anchoring of active p38 MAPK to its target gene *Hspb1* may be mediated by the transcription factor p53 (Fig. 6).

Our current data demonstrate that p-HSPB1 is upregulated in livers and spleens in which amyloid deposition was mitigated, and it accumulates at sites of amyloid deposition. Thus, we suggest that the induction of p-HSPB1 could be a novel mechanism by which exercise reduces amyloidosis. HSPB1 can be elevated in the brain and accumulate in senile plaques in AD patients (Shinohara et al., 1993; Renkawek et al., 1994). Moreover, overexpression of *Hspb1* mitigates Aβ deposition and cognitive dysfunction in a mouse model of AD (Toth et al., 2013). Recent *in vitro* evidence has suggested that when HSPB1 undergoes phosphorylation under stress conditions, its chaperone activity is enhanced by increasing its binding affinity for client amyloid proteins, including Aβ, alpha-synuclein, microtubule-associated protein tau and RNA-binding protein FUS, thereby inhibiting formation from both amyloid and amorphous aggregation (Jovcevski et al., 2015; Nafar et al., 2016; Baughman et al., 2020; Liu et al., 2020; Selig et al., 2020). Structural studies demonstrated that HSPB1 can recognize the motifs enriched in hydrophobic or low-complexity regions in the peptides of client proteins, reducing aggregation (Baughman et al., 2018; Janowska et al., 2019). Our previous study demonstrated that the hydrophobic 11-residue peptide at the N terminus and 18-residue peptide at the C terminus of APOA-II protein are indispensable for polymerization into amyloid fibrils, and may constitute binding regions of HSPB1 (Sawashita et al., 2009).

We previously found that the mRNA levels of UPR/ER stress-related genes were elevated in the livers of mice with AApoAII amyloidosis (Luo et al., 2015). Here, we found that UPR was significantly upregulated in the livers of sedentary mice with amyloidosis (Fig. 7). It was previously reported that UPR activates p38 MAPK through the classical MAPK kinase (MKK)3/MKK6 upstream kinase cascade (Matsuzawa et al., 2002). Moreover, it was suggested that the p38 MAPK signaling pathway has positive effects on ER homeostasis via a complex feedback loop regulating UPR signaling elements (Lee et al., 2011). Interestingly, amyloid deposition did not significantly increase the phosphorylation of HSPB1. Nevertheless, activated p38 MAPK was observed. It is not clear why the activation of p38 MAPK by amyloid deposition failed to increase the phosphorylation of HSPB1. However, it is known that p38 MAPK-induced HSPB1 phosphorylation is dependent on the activation of MAPK activated protein kinase 2 (MK2) (Stokoe et al., 1992; Zarubin and Han et al., 2005). Moreover, inhibition of MK2 in hepatocytes or livers of mice suppresses HSPB1 phosphorylation (Ozcan et al., 2015). In addition, other protein kinases can phosphorylate HSPB1, including cyclic AMP-dependent protein kinase A, protein kinase C and protein kinase D (Kostenko and Moens et al., 2009). The activation of these kinases, along with MK2, has a demonstrated association with exercise (Krook et al., 2000; Rose et al., 2004; Williamson et al., 2006; Ellwanger et al., 2011).

Heat shock proteins (HSPs) are involved in both intracellular and extracellular protein homeostasis by chaperoning the misfolded proteins during ER stress (Genereux et al., 2015). In systemic ATTRmt amyloidosis, extracellular amyloid deposition induces higher expression of HSPB1 and HSP70 in the peripheral nerves, skin and salivary glands of the patients (Santos et al., 2008). Studies reported overexpression of HSPB1, CRYAB and HSP70 in the brains of AD and Parkinson's disease patients (Shinohara et al., 1993; Renkawek et al., 1999). However, our data showed that IT and amyloidosis induced upregulation of *Hspb1* and HSPB1 in the liver and spleen but did not increase other common HSPs, such as *Hsp70*/*Hspa1b* and *Cryab* (also known as *Hspb5*). Although transcription factor HSF1 is generally responsible for transcriptional regulation of *Hspb1* and other HSPs (Benarroch, 2011; Kourtis and Tavernarakis et al., 2011), we suggest that the higher expression of *Hspb1* is independent from HSF1 and is specifically regulated by other transcription factors, including TP53. There is recent evidence from the retinal ischemia rat model that HSPB1 (rather than other common HSPs) is the transcriptional target of the hypoxia-inducible factor (HIF)-1α transcription factor (Whitlock et al., 2005). Given that exercise increases oxygen consumption and reduces intracellular oxygen partial pressure, this stress could contribute to HIF-1α activation (Lindholm and Rundqvist et al., 2016).

Here, we used two exercise regimens. IT for mice mimics the human IWT, an exercise program at submaximal intensity that combines aerobic and resistance training. The molecular mechanisms underlying this regimen have not been elucidated because there were no appropriate animal models. Another exercise regimen, CT, uses aerobic/endurance training at moderate intensity. We observed that both IT and CT increased muscle mass, improved glucose intolerance and several physiological functions. We also observed that both IT and CT upregulated myokine *Il6* expression in the muscle after exercise, but the effect of IT was more significant. Interestingly, both exercise regimens had similar positive effects on systemic amyloidosis. We assume that the adaptive response to a CT regimen could be sufficient to suppress the progression of AApoAII amyloidosis. In other age-related diseases, such as type 2 diabetes, 4-month resistance training or IWT training is more effective at improving pathological conditions and physical fitness (Cauza et al., 2005; Karstoft et al., 2013). Although strong evidence supports the benefits of endurance exercise in improving the cognitive abilities of AD patients, the effect of resistance exercise remains unclear (Panza et al., 2018; Herold et al., 2019).

In conclusion, to our knowledge, this is the first demonstration of one possible mechanism in which exercise additively enhances the expression of HSPB1 in the presence of amyloid deposition, and activates the anti-amyloid activity of HSPB1 (p-HSPB1), which prevents amyloidosis *in vivo*. Although further investigation is needed to better characterize the participating molecular pathways, we suspect that exercise can regulate signaling programs that enhance the expression of appropriate adaptive molecules in the presence of harmful extracellular or intracellular conditions (such as amyloidosis). Our findings suggest a biochemical basis that explains how exercise reduces the risk of age-related disorders. New therapeutic strategies should build upon these findings, further improving treatment strategies.

## MATERIALS AND METHODS

### Animals and experimental design

Female R1.P1-*Apoa2^c^* congenic mice that have a normal aging process were used in this study. This strain carries the amyloidogenic *Apoa2^c^* allele from the AApoAII amyloidosis-susceptible senescence-accelerated mouse prone 1 (SAMP1) strain on a genetic background of the senescence-accelerated mouse resistant 1 (SAMR1) strain (Higuchi et al., 1995). AApoAII amyloidosis in both male and female R1.P1-*Apoa2^c^* mice can be induced systemically and quantitatively by intravenous injection of a small amount of AApoAII amyloid fibrils (Xing et al., 2001). The mice were maintained under specific pathogen-free (SPF) conditions at 24±2°C with a light-controlled regimen (12 h light/dark cycle), and were fed a commercial diet (MF, Oriental Yeast, Tokyo, Japan) with tap water given *ad libitum* in the Division of Animal Research, Research Center for Supports to Advanced Science, Shinshu University.

In terms of experimental design (Fig. 1), a series of three independent experiments involving a total of 79 mice were repeated at different times. In each experimental series, 8-week-old female mice with similar body weights were randomly divided into sedentary, IT and CT groups (Table S1, Fig. S1). Before the exercise training (pre-check), each mouse was characterized for its maximal running speed (V_max_) and various physiological indices, including 24-h voluntary activity, body temperature, 15-h fasting body temperature, heart rate, blood pressure and intraperitoneal glucose tolerance (IGTT). Then, 41 10-week-old mice were injected intravenously with 1 μg/mouse of AApoAII fibrils to induce amyloidosis. The remaining 38 10-week-old mice were injected with PBS. The mice then undertook exercise regimens for 16 weeks. After 8 weeks of exercise, the V_max_ of all mice was re-measured and re-calculated, and the running speed of each mouse in the IT and CT groups was maintained until the end of the training period. Additionally, 2 weeks before the end of the exercise training (post-check), the V_max_ and above-mentioned physiological indices were measured again. Lastly, 63 mice were selected for further analyses, including physiological and molecular biological determinations based on the exclusion criteria, including: (1) presence of injuries, abscesses or other signs of illness; (2) running avoidance behaviors more than three consecutive times (i.e. less than 10 min at each exercise interval) for mice in the IT and CT groups; and (3) unexpected death (Table S1, Fig. S1). Experiments with mice were performed with the approval of the Committee for Animal Experiments of Shinshu University (approval number 290007), and approved protocols were strictly adhered to.

### Treadmill exercise protocol

#### Determination of the maximal running speed

Mice were acclimated to the treadmill (TMS-4, Melquest Corp, Japan) three times (15 min at 10 m/min) on different days prior to determination of the V_max_ for each mouse. The test started at 10 m/min for 20 s, followed by a continued stepwise increase (1 m/min) in running speed every 20 s until exhaustion. Exhaustion was indicated when the mice fell back on the electric shock bar three times within 30 s rather than running on the treadmill. V_max_ was determined as the last completed stage during the incremental test (e.g. if the mouse could not run at 40 m/min, then the V_max_ was 39 m/min). The test was performed at 0 weeks (pre-check), 8 weeks (middle-check) and 16 weeks (post-check) during the 16-week exercise intervention.

#### High-intensity interval training protocol

IT training was performed 4 days per week on a treadmill based on a protocol modified from that described for a human study (Masuki et al., 2020). Each training session was preceded by a 3-min warm-up with running at 10 m/min. The 30-min training sessions involved five sets of 3-min low-intensity running intervals at a speed that was 30% of the pre-check V_max_ followed by 3 min of high-intensity running at a speed that was 70% of the pre-check V_max_. After 8 weeks of training, the V_max_ was checked again and the training cycles were adjusted accordingly. The adjusted V_max_ values were used through the remaining 8 weeks of the 16-week training period.

#### Moderate-intensity continuous training protocol

CT training was performed 4 days per week on a treadmill based on a protocol that was slightly modified from a previous study (He et al., 2012). For CT, the above-mentioned warm-up was used and then the mice undertook continuous running for 30 min at a speed that produced 50% of the pre-check V_max_. After 8 weeks of training, the V_max_ was adjusted as described above.

### Induction of AApoAII amyloidosis

AApoAII amyloid fibrils were isolated from the livers of R1.P1-*Apoa2^c^* mice with severe amyloid deposition using a modified Pras method as described previously (Pras et al., 1969). Mice in the amyloidosis-induced groups (FS, FI and FC) were injected intravenously at 10 weeks of age with 1 μg AApoAII fibrils in 100 μl PBS to induce AApoAII amyloidosis. AApoAII fibrils were sonicated on ice according to our previous method (Xing et al., 2001) before injection.

### Detection of amyloid deposition in mice

The main organs were fixed in 10% neutral buffered formalin, then embedded in paraffin and cut into 4-μm sections using standard procedures. Amyloid deposition was identified using polarizing light microscopy (Axioskop 2, Carl Zeiss Japan, Tokyo, Japan) to observe apple-green birefringence in tissue sections stained with Congo Red (Sawashita et al., 2009). The degree of amyloid deposition formation in examined organs was scored from 0 to 4 (amyloid score) according to a previously described scoring principle (Higuchi et al., 1998). The systemic degree of amyloid deposition in each mouse was determined semi-quantitatively using an amyloid index that represents the average of amyloid scores for seven organs (heart, liver, spleen, tongue, stomach, small intestine and skin) (Higuchi et al., 1998).

### Measurements of physiological indices and serum lipid parameters

We determined physiological indices, including 24-h voluntary activity, body temperature, 15-h fasting body temperature, heart rate, blood pressure and IGTT for each mouse at two timepoints: before training (when mice were 8 weeks old) termed the pre-check, and 2 weeks before the end of the 16-week training (when mice were 24 weeks old), termed the post-check. Twenty four hours voluntary activity was measured with a wheel cage (SN-450, Shinano Ltd., Tokyo, Japan). Both body temperature and 15-h fasting temperature were measured using a 10 s flexi tip thermometer (ST8731CS, MSR Tech. Co. Ltd., China). Heart rate and blood pressure were measured using a computerized tail-cuff system (BP-98A-L, Softron Ltd., Tokyo, Japan). For IGTT, mice that had fasted for 15 h were injected intraperitoneally with glucose (2* *g/kg body weight). Tail vein blood samples were obtained at 0, 15, 30, 60, 90 and 120 min. Glucose levels were measured with a handheld glucose meter (Accu-Check Aviva, Roche Diagnostic, Tokyo, Japan). Serum total- and HDL-cholesterol levels, as well as triglyceride (TG) levels, were determined using quantitative assay kits (Total-cholesterol E test, 439-17501; HDL-cholesterol E test, 431-52501; TG E test, 432-40201, Fujifilm Wako, Osaka, Japan).

### Immunohistochemical and immunofluorescent analysis

AApoAII deposition and phospho-HSPB1 were evaluated by IHC using the horseradish peroxidase-labeled streptavidin-biotin method with specific antibodies. Rabbit polyclonal antiserum against mouse ApoA-II was produced in our laboratory (Higuchi et al., 1997) and applied at a dilution of 1:3000. Rabbit monoclonal anti-phospho-HSPB1 (Ser82) antibody (9709T, Cell Signaling Technology) was applied (1:100) to the paraffin-embedded sections. After incubation with the primary antibody overnight at 4°C, sections were incubated with a biotinylated secondary anti-rabbit IgG antibody (1:300, E0353, Dako) for 1 h at room temperature before application of horseradish peroxidase-labeled streptavidin-biotin (1:300, P0397, Dako) to detect target proteins. For immunofluorescence analyses, paraffin-embedded sections were incubated with TP53 rabbit polyclonal antibody (1:50, 10442-1-AP, Proteintech) overnight at 4°C, incubated with Alex Fluor 594 goat anti-rabbit IgG antibody (1:500, R37117, Thermo Fisher Scientific) for 1 h at room temperature, and then incubated with DAPI for 30 min. Images were captured immediately using a confocal laser fluorescence microscope (LSM 880 with Airyscan, Zeiss, Germany). For negative control sections, the primary antibody was omitted to confirm staining specificity. For quantitative analysis of amyloid deposition, five areas in each liver and spleen section were randomly captured under 200× magnification, and the ratios of areas positively stained with anti-APOA-II antiserum to whole-section areas were calculated using an image processing program [ImageJ, version 1.61, National Institutes of Health (NIH), Bethesda, MD, USA].

### Immunoblot analysis

Protein levels were measured by western blotting analysis as described previously (Li et al., 2017; Dai et al., 2019). Proteins were extracted from liver, spleen and lungs using the RIPA Lysis Buffer System (Santa Cruz Biotechnology, Dallas, TX, USA), and protein concentrations were measured using a BCA Protein Assay Kit (Thermo Fisher Scientific, Rockford, IL, USA). Serum samples (0.5 μl) were used to detect APOA-II and APOA-I, and liver lysates (50 μg protein) were used to detect phospho-p38 MAPK, total p38 MAPK, TP53, PGC1-α, GRP78/BIP and β-actin. Phospho-HSPB1 and total HSPB1 were detected in lysates of liver, spleen and lung (100 μg protein). Proteins were separated using either 16.5% (APOA-II and APOA-I) or 12% SDS-PAGE (all other proteins). The following antibodies and dilutions were used for detection: anti-APOA-II [produced in our lab (Higuchi et al., 1997), 1:3000]; anti-APOA-I [produced in our lab (Li et al., 2017), 1:5000]; anti-HSPB1 (1:500, GTX101145, GeneTex); anti-phospho-HSPB1 (Ser82) (1:500, 9709, Cell Signaling Technology); anti-p38 MAPK (1:1000, GTX110720, GeneTex); anti-phospho-p38 MAPK (Thr180/Tyr182) (1:1000, GTX113460, GeneTex); anti-TP53 (1:1000, 10442-1-AP, Proteintech); anti-PGC1-α (1:200, SC-13067, Santa Cruz Biotechnology); anti-BIP (1:200, SC-1050, Santa Cruz Biotechnology); and β-actin (1:1000, AP0060, Bioworld). After electrophoresis, proteins were transferred to a polyvinylidene difluoride membrane (Immobilon, 0.2μm pore, MerckMillipore, Burlington, MA, USA). The membranes were blocked with 5% bovine serum albumin or non-fat dry milk in TBS according to the antibody manual for 1 h at room temperature, and incubated with primary antibodies overnight at 4°C. Horseradish peroxidase-conjugated anti-rabbit IgG (1:3000, 7074, Cell Signaling Technology) was then applied as the secondary antibody and incubated with the membranes for 1 h at room temperature. Protein bands were detected by enhanced chemiluminescence, and the target proteins were analyzed using NIH ImageJ software.

### Liver RNA-seq analysis

Although both IT and CT effectively suppressed AApoAII amyloid deposits in the liver to similar degrees, we selected liver samples from the IT groups for RNA-seq analysis because IT stimuli offered more metabolic benefits in skeletal muscle at a transcriptional level. Briefly, 10-mg liver samples taken from each mouse and stored at −80°C were homogenized in TRIzol RNA isolation reagent (Invitrogen, Thermo Fisher Scientific, Tokyo, Japan), and then pooled in four sample tubes (VS, VI, FS and FI groups, *N*=4). The sample tubes were sent to Novogene (Nagoya, Japan) for analysis. Total RNA was extracted, and RNA purity and integrity were confirmed by Novogene. Sequencing analysis of expressed RNAs was performed using an Illumina next generation sequencing platform. Sequencing count data were analyzed using edgeR R package (3.4) software to identify significant DEGs among the different groups. Corrected *P*<0.05 and absolute fold-change >1 were set as thresholds for DEGs. Biological functions for the accumulated DEGs were analyzed using an annotation database (GO and KEGG Pathway Database) to elucidate mechanisms associated with beneficial effects of interval exercise. GO and KEGG pathways enrichment analyses of DEGs were performed using the clusterProfiler R package. GO terms and KEGG pathways with corrected *P*<0.05 were considered significantly enriched by DEGs. GSEA was performed using the Java GSEA implementation. The gene lists of GO or KEGG gene signature were adopted from The Molecular Signatures Database (MSigDB).

### Gene expression analysis with real-time qPCR

Analysis of mRNA expression in the liver, skeletal muscle, spleen and lung was performed as described previously (Dai et al., 2019). Total RNA was extracted from quick-frozen samples using TRIzol Reagent (Invitrogen, Carlsbad, CA, USA). Total RNA (10 ng/μl final concentration) was reverse-transcribed using a High Capacity cDNA Reverse Transcription Kit with random primers (Applied Biosystems, Fisher Scientific, Tokyo, Japan). Real-time qPCR analysis was carried out using a sequence detection system (Abi Prism 7500, Applied Biosystems, Foster City, CA, USA) with SYBR Green (TaKaRa Bio, Tokyo, Japan). Gene expression was normalized relative to 18S ribosomal RNA. The forward and reverse primer sequences are listed in Table S3.

### TUNEL assay for apoptosis detection

Formalin-fixed paraffin-embedded tissue blocks were cut into 4-mm sections and processed for use in terminal deoxynucleotidyl transferase-mediated dUTP nick-end labeling (TUNEL) assays (Luo et al., 2015). Sections were stained using an *In Situ* Apoptosis Detection Kit (TaKaRa, MK500, Japan), according to the manufacturer's instructions. TUNEL-positive cells in tissues were counted in three fields per section at 200-fold and 400-fold magnifications, using light microscopy.

### Reproducibility of results and statistical analysis

We repeated three independent series at different periods (first series, May 2017; second series, October 2017; third series, December 2018) to determine the effects of 16-week exercise training on inhibition of amyloidosis. In each series, we measured various physiological indices before and after training, and performed IHC and Congo Red staining for quantitative analysis of AApoAII amyloid deposition. Molecular biochemical experiments (western blot, real-time qPCR and immunostaining) were performed two times for reproducibility.

For comparison of parametric data, ANOVA was performed using the SPSS 26.0 software package (Abacus Concepts, Berkley, CA, USA). One-way ANOVA was used to compare sedentary and exercise groups. Two-way ANOVA was used to compare the magnitude of changes between different groups of mice with or without amyloidosis induction. Repeated-measures ANOVA was used to compare the magnitude of changes within groups before and after training. For comparison of non-parametric data, the Kruskal–Wallis test with the Steel–Dwass test was used to analyze the averages of amyloid score and AI for amyloid deposition among groups using R software version 3.4.3. *P*<0.05 was considered statistically significant.

## Supplementary Material

Supplementary information
