# Supplementary material for: Exercise suppresses mouse systemic AApoAII amyloidosis through enhancement of the p38 MAPK signaling pathway
Source: Dis Model Mech. 2022 Mar 21;15(3):dmm049327. doi: 10.1242/dmm.049327 (PMC8961676; doi:10.1242/dmm.049327)
Supplement: Supplementary information [file dmm-15-049327-s1.pdf]

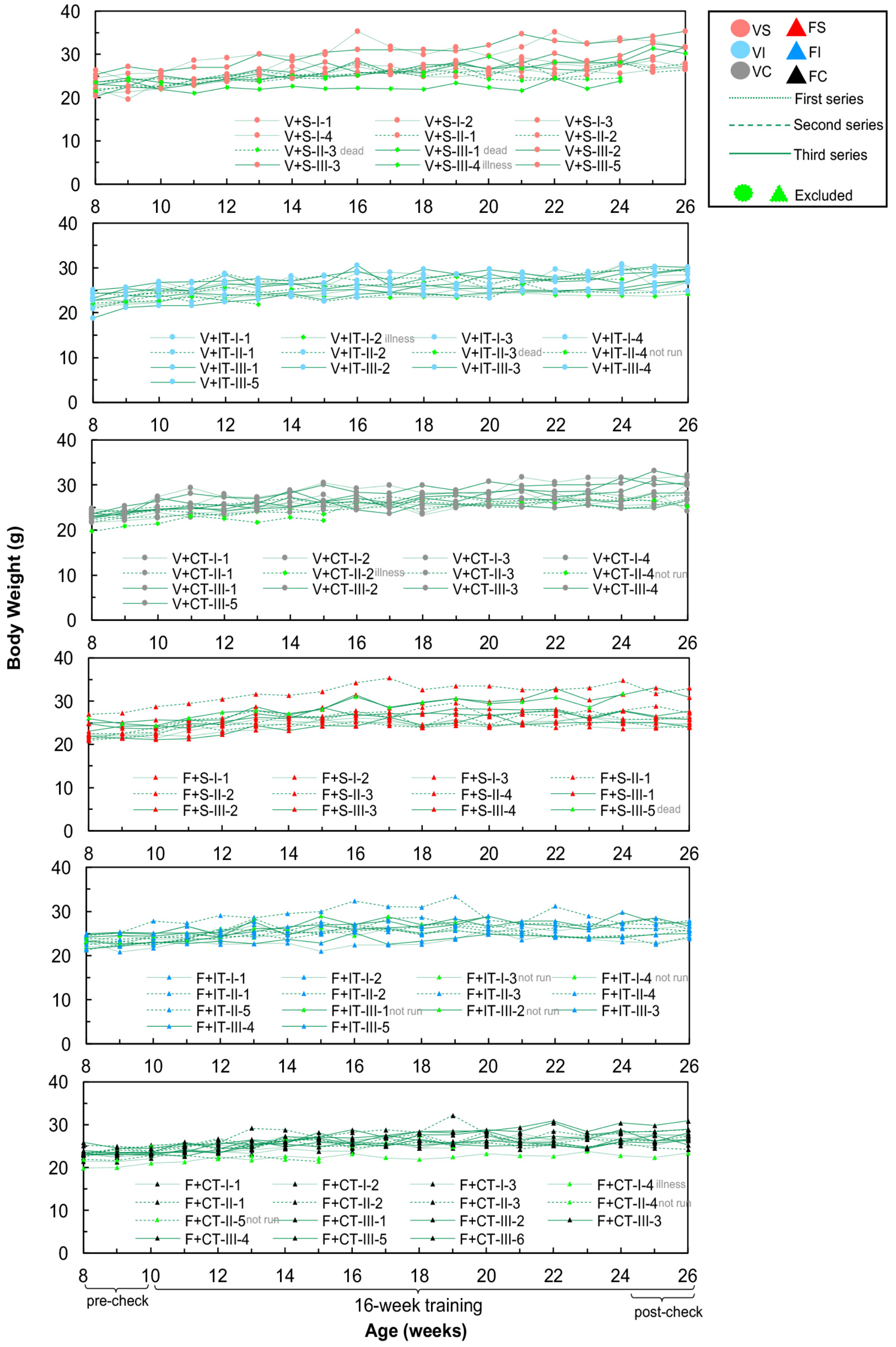

Fig. S1. Body weight of prepared mice: raw data
Three independent experimental series involving 79 mice were conducted **(Table S1).** No significant difference in body weights among any of the six experimental groups from the start to the end of 16 weeks of exercise treatment. Based on the exclusion criteria described in the Materials and Methods, 63 mice were selected for physiological analyses.

Disease Models & Mechanisms • Supplementary information

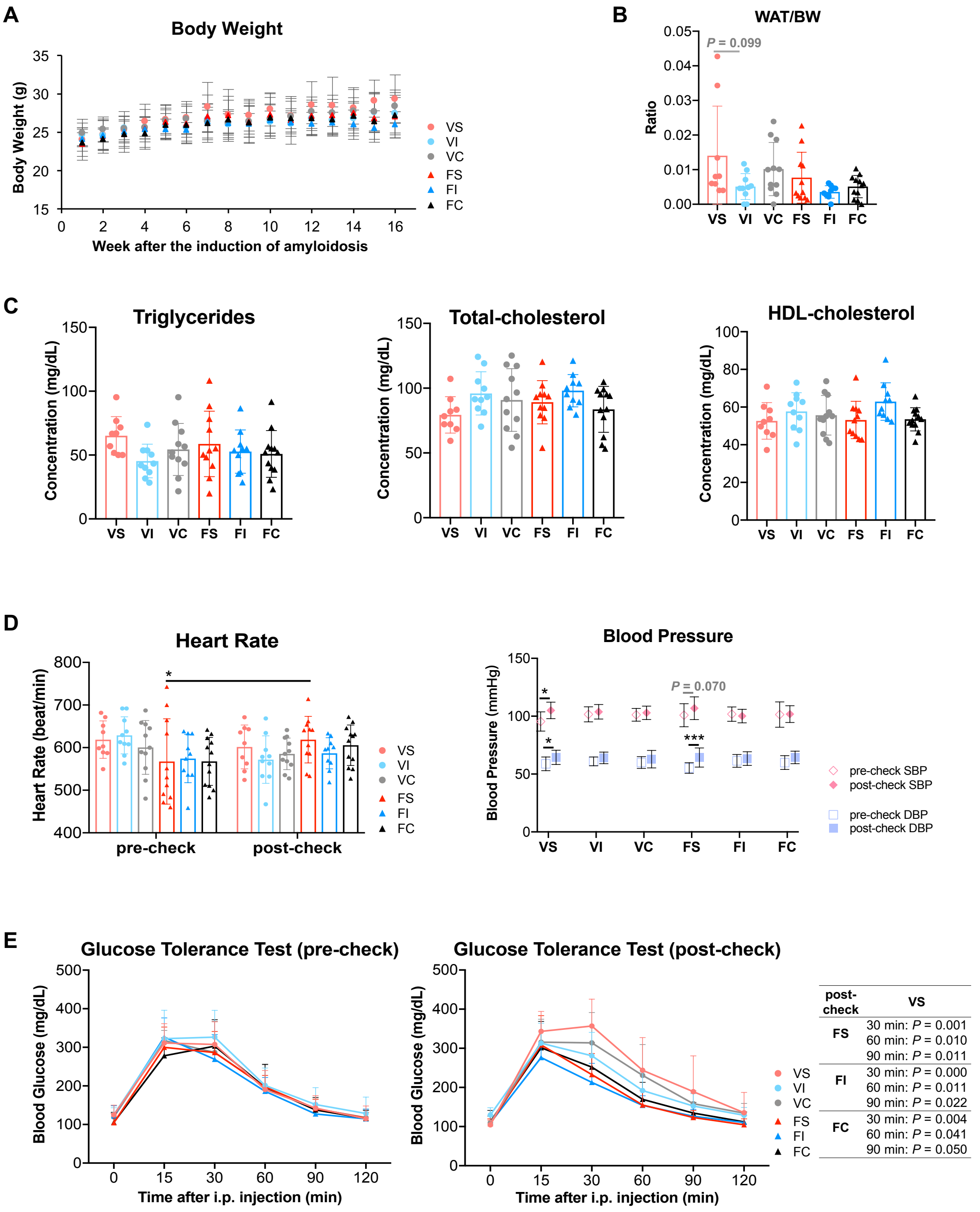

Fig. S2. Physiological indices in pre-check and post-check

(A) Body weights during the 16-week exercise and AApoAII amyloidosis induction. **(B)** Ratio of WAT weight to body weight after the 16-week interventions. **(C)** Serum lipid profile after the 16-week interventions. **(D)** Heart rates and blood pressures before and after interventions. **(E)** Intraperitoneal glucose tolerance test before and after interventions. Each dot represents an individual mouse (B-D). Data represent means ± SD (VS, *n* = 9; VI, *n* = 10; VC, *n* = 11; FS, *n* = 11; FI, *n* = 10; FC, *n* = 12). $^{\#}P < 0.05$; $^{\#\#}P < 0.01$; $^{\#\#\#}P < 0.001$ (one-way ANOVA with Tukey-Kramer method for comparison between groups). $^{*}P < 0.05$ vs. respective pre-check (Repeated-measures ANOVA).

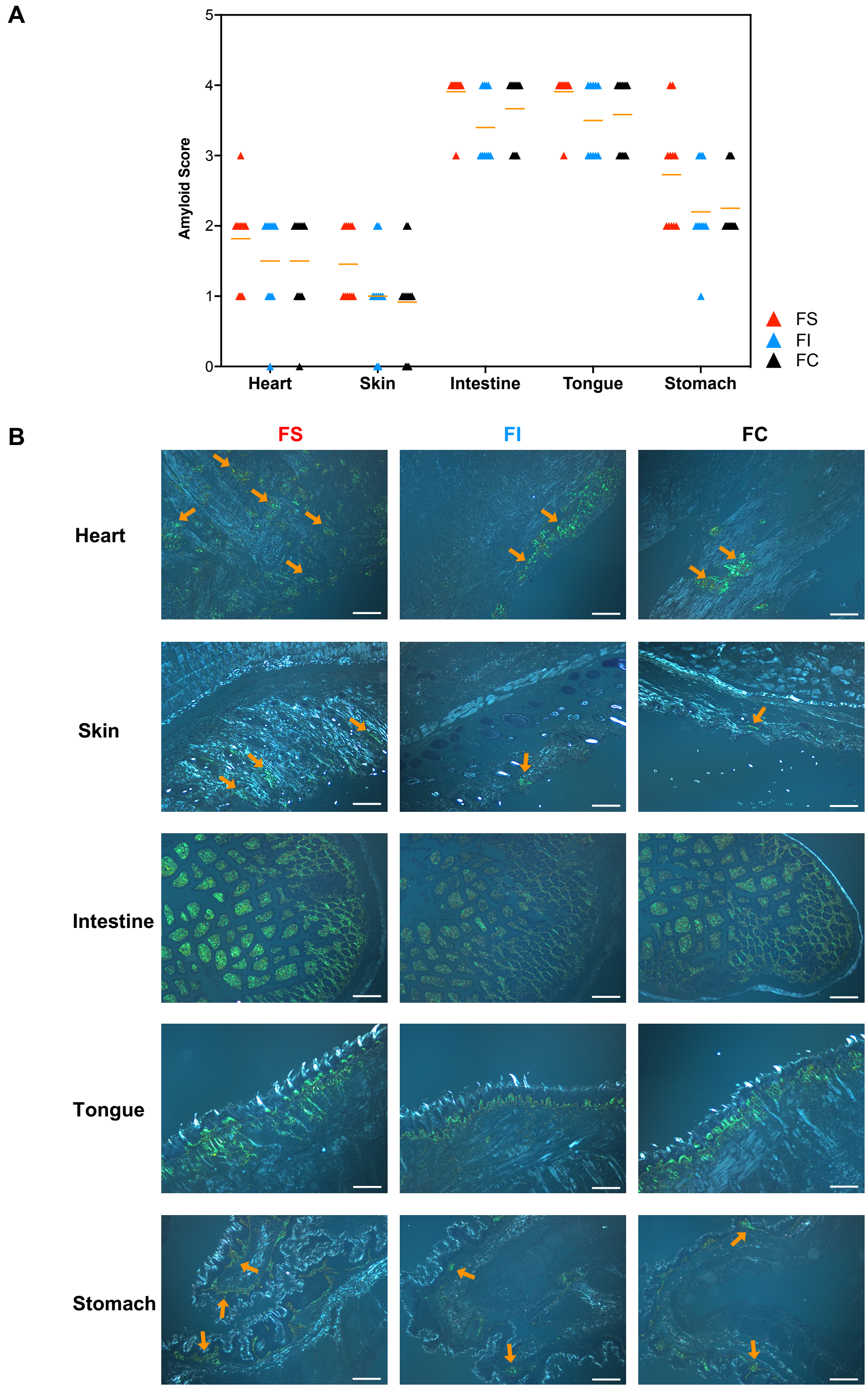

Fig. S3. Amyloid scores in examined organs other than liver and spleen
(A) Amyloid scores for the heart, skin, intestine, tongue, stomach in the fibril groups. Each dot represents an individual mouse (FS, *n* = 11; FI, *n* = 10; FC, *n* = 12). **(B)** Representative Congo Red and apple-green birefringent images of amyloid deposition (orange arrows) of the indicated organs from amyloidosis-induced mice. Scale bars indicate 100 µm.

Disease Models & Mechanisms • Supplementary information

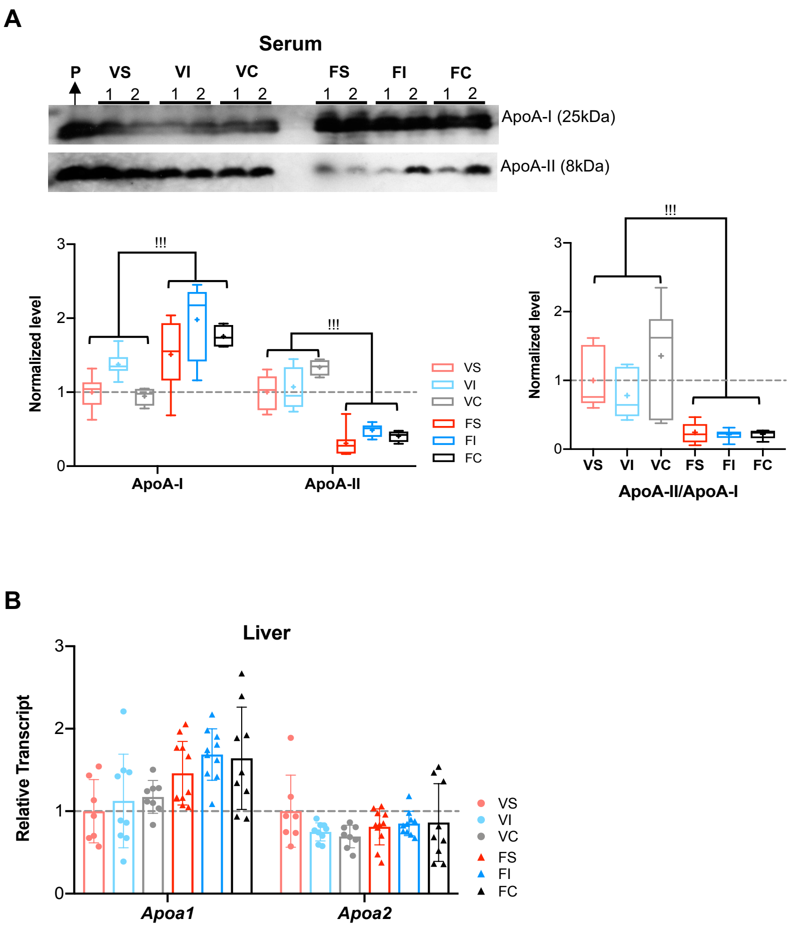

Fig. S4. Exercise did not affect serum levels of precursor protein
(A) Western blot and quantification of ApoA-I and ApoA-II in sera of mice after 16 weeks of interventions (VS, *n* = 9; VI, *n* = 10; VC, *n* = 6; FS, *n* = 10; FI, *n* = 10; FC, *n* = 6). P (positive control) indicates the pooled sera of 8-week-old mice (*n* = 4) without AApoAII amyloidosis. **(B)** *Apoa1* and *Apoa2* mRNA expression levels in livers of mice after 16 weeks of interventions normalized to 18S (VS, *n* = 7; VI, *n* = 9; VC, *n* = 8; FS, *n* = 11; FI, *n* = 10; FC, *n* = 9). Each dot represents an individual mouse. Data represent means ± SD. $^{!!!}P < 0.001$ (two-way ANOVA for comparison of the magnitude of changes between different groups in mice with or without amyloidosis induction).

Disease Models & Mechanisms • Supplementary information

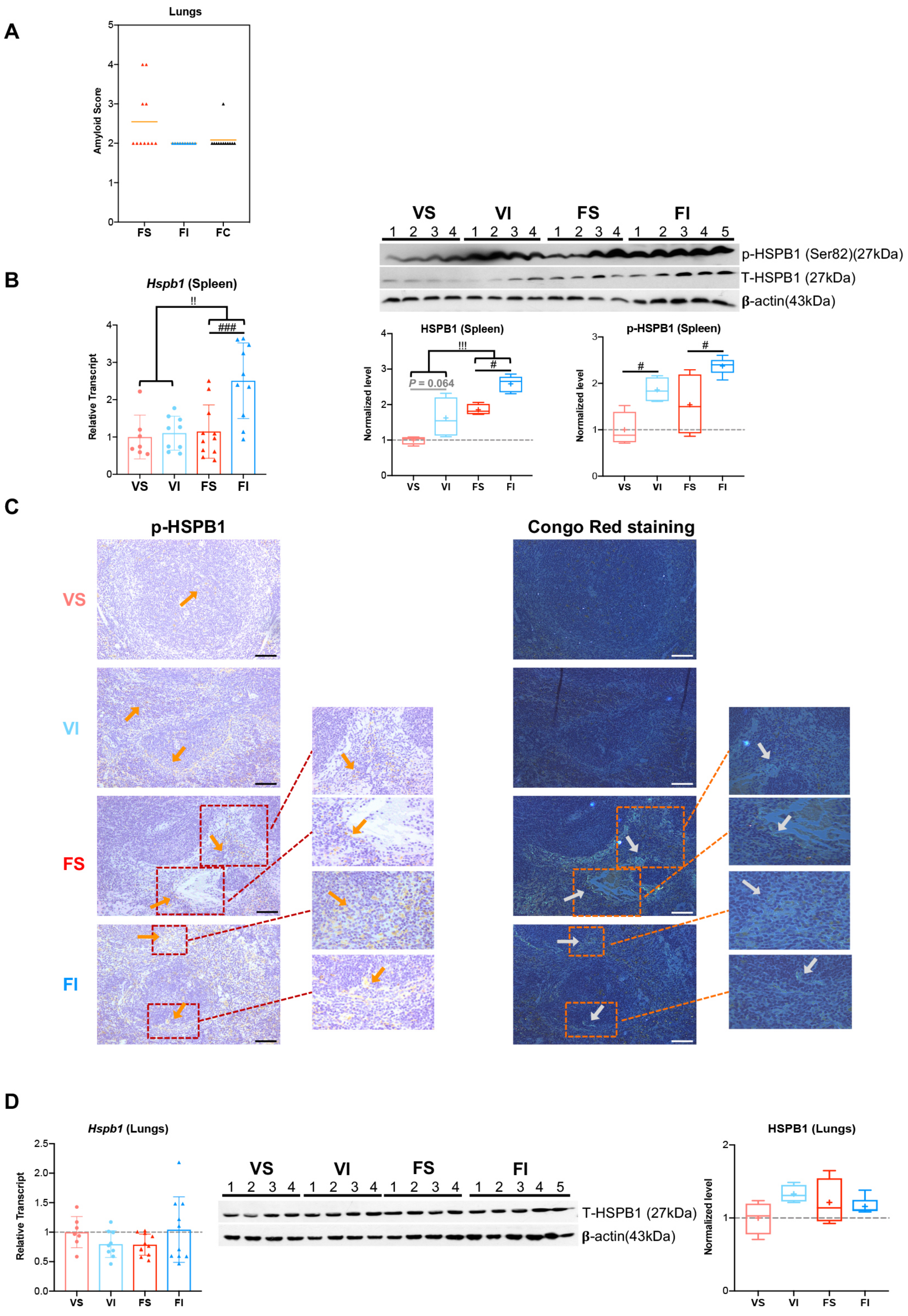
A
Lungs
Amyloid Score
FS
FI
FC
B
Hspb1 (Spleen)
Relative Transcript
VS
VI
FS
FI
VS VI FS FI
p-HSPB1 (Ser82)(27kDa)
T-HSPB1 (27kDa)
β-actin(43kDa)
HSPB1 (Spleen)
p-HSPB1 (Spleen)
Normalized level
P = 0.064
C
p-HSPB1
Congo Red staining
VS
VI
FS
FI
D
Hspb1 (Lungs)
Relative Transcript
T-HSPB1 (27kDa)
β-actin(43kDa)
HSPB1 (Lungs)
Normalized level

Fig. S5. IT-dependent increased p-HSPB1 might play a protective role against amyloidosis in spleen

(A) Amyloid scores (FS, *n* = 11; FI, *n* = 10; FC, *n* = 12) of the lungs of amyloidosis-induced mice. **(B)** mRNA expression of *Hspb1* normalized to 18S in the spleen after 16 weeks of the indicated exercise and amyloidosis induction (VS, *n* = 7; VI, *n* = 9; FS, *n* = 11; FI, *n* = 10). Western blot and quantification of total-HSPB1 and p-HSPB1 in the spleens of mice after the 16-week interventions (*n* = 4). **(C)** Representative images of IHC with anti-p-HSPB1 antibody and Congo Red staining of amyloid deposition in the spleen of mice after 16-week interventions. p-HSPB1 (orange arrows) was confirmed by IHC. Amyloid deposits (gray arrows) were identified by apple-green color birefringence in Congo Red-stained sections under polarizing light microscopy. Scale bars indicate 100 μm. **(D)** *Hspb1* mRNA expression normalized to 18S in the lungs of mice after 16 weeks of indicated exercise and amyloidosis induction (VS, *n* = 7; VI, *n* = 9; FS, *n* = 11; FI, *n* = 10). Western blot and quantification of total-HSPB1 in the lungs of mice after 16-week interventions (*n* = 4). Each dot represents an individual mouse (A, B, D). Data represent means ± SD. $^{\#}P < 0.05$; $^{\#\#\#}P < 0.001$ (one-way ANOVA with Tukey-Kramer method for comparison between groups). $^{!!}P < 0.01$; $^{!!!}P < 0.001$ (two-way ANOVA to compare the magnitude of changes between different groups of mice with or without amyloidosis induction).

Disease Models & Mechanisms • Supplementary information

A

RNA seq	Read count				corrected *P* value	
DEGs	VS	VI	FS	FI	VI vs. VS	FI vs. FS
Gadd45g	157	800	1792	3837	0.0000	0.0000
Cdkn1a	133	863	908	1356	0.0000	0.0022

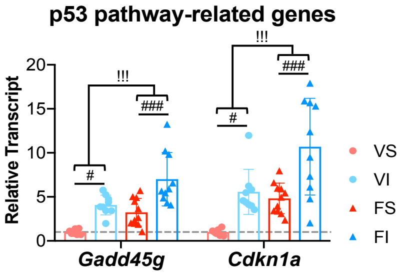

B

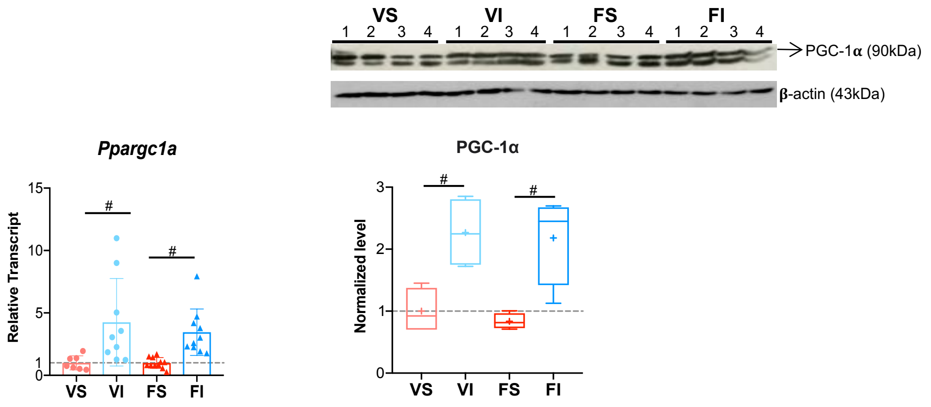

C

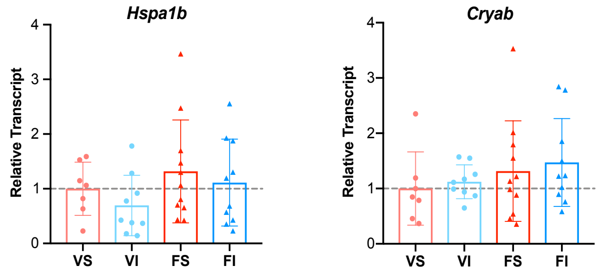

Fig. S6. IT induced higher levels of p53 pathway-related genes

(A) RNA sequencing analysis of DEGs indicated *Gadd45g* and *Cdkn1a* in p53 signaling pathway. Real-time qPCR of mRNA levels of p53 signaling pathway-related *Gadd45g* and *Cdkn1a* genes normalized to 18S in the livers of mice after 16-week interventions. **(B)** Real-time qPCR and Western blot (n = 4) analysis of PGC1-α in the livers of mice after 16-week interventions. **(C)** Real-time qPCR of HSF1-dependent *Hspa1b* and *Cryab* mRNAs normalized to 18S in the livers of mice after 16-week interventions. Each dot represents an individual mouse (A-C) (VS, n = 7; VI, n = 9; FS, n = 11; FI, n = 10). Data represent means ± SD. $^{\#}P < 0.05$; $^{\#\#\#}P < 0.001$ (one-way ANOVA with Tukey-Kramer method for comparison between groups). $^{!!!}P < 0.001$ (two-way ANOVA to compare the magnitude of changes between different groups of mice with or without amyloidosis induction).

Disease Models & Mechanisms • Supplementary information

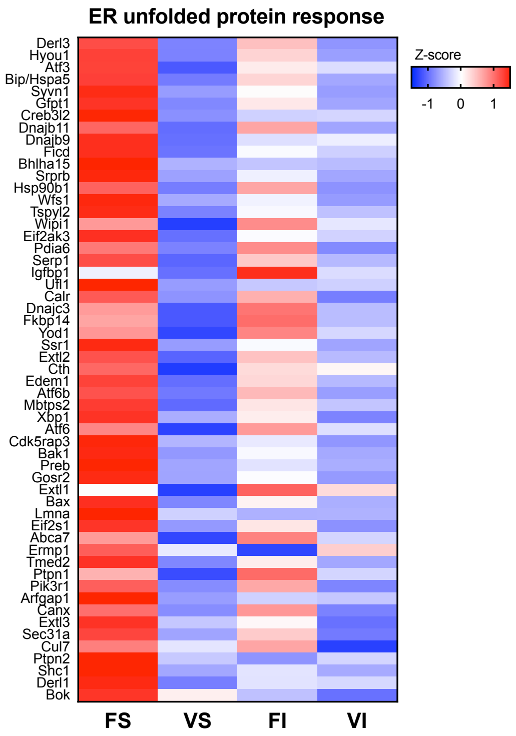

Fig. S7. ER stress responses in the livers of amyloidosis-induced mice
Heat-map analysis of gene-set in ER stress identified by GSEA.

Disease Models & Mechanisms • Supplementary information

RNA seq	Read count				corrected *P* value	
DEGs	VS	VI	FS	FI	VI vs. VS	FI vs. FS
Ppargc1a	259	485	518	619	0.0105	0.1399
Pdk4	613	1242	171	1077	0.0006	0.0000
G6pc	4875	9290	5349	16799	0.0014	0.0000

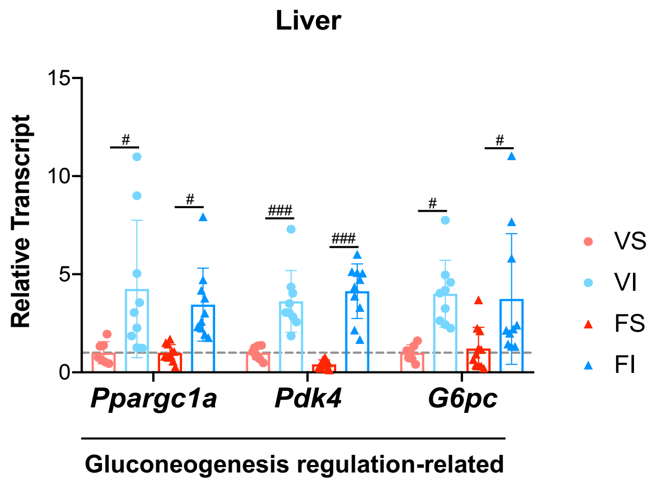

Fig. S8. IT induced higher expression of genes related to gluconeogenesis in the liver

Data of read counts and significance of DEGs involved in hepatic gluconeogenesis based on RNA-seq. Real-time qPCR analysis of mRNA levels of gluconeogenesis-related genes (*Ppargc1a, Pdk4 and G6pc*) normalized to 18S in the livers of mice after 16-week interventions. Each dot represents an individual mouse. Data represent mean ± SD (VS, n = 7; VI, n = 9; FS, n = 11; FI, n = 10). $^{\#}P < 0.05$; $^{\#\#\#}P < 0.001$ (one-way ANOVA with Tukey-Kramer method for comparison between groups).

Disease Models & Mechanisms • Supplementary information

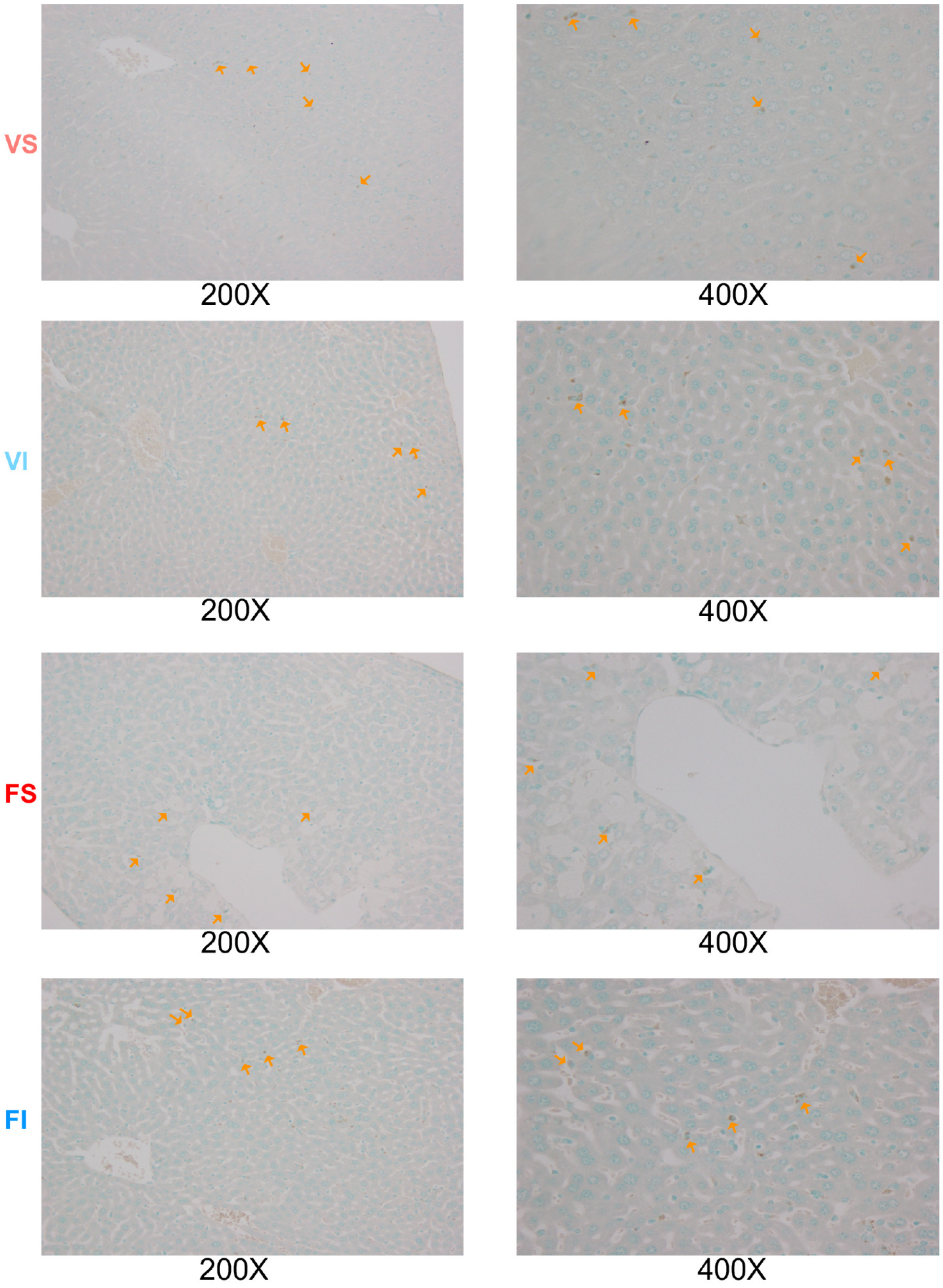

Fig. S9. Similar numbers of apoptosis-positive cells in livers from mice in different experimental groups after 16 week interventions.
Apoptosis-positive cells in livers were similar among groups after 16-week interventions. Representative images of TUNEL-positive cells (indicated by orange arrows) in the liver at 200x and 400x magnification.

Table S1. Raw data for prepared and examined mice in three series.

Group	Prepared mice		Tested mice	Excluded mice					
	Number	BW (g) 8-week-old	Number	Death		Dropout		Other	
				Number	Reason	Number	Reason	Number	Reason
**First Series: Birthday: 9-11 May in 2017**									
V+S	4	23.47±2.39	4						
V+IT	4	22.33±0.96	3					1	illness
V+CT	4	23.53±1.34	4						
F+S	3	21.90±0.47	3						
F+IT	4	22.96±0.78	2			2	Not run		
F+CT	4	21.99±1.65	3					1	illness
**Second Series: Birthday: 25-29 October in 2017**									
V+S	3	21.71±0.36	2	1	Unknown				
V+IT	4	22.57±1.53	2	1	Unknown	1	Not run		
V+CT	4	21.89±1.69	2			1	Not run	1	illness
F+S	4	22.88±2.81	4						
F+IT	5	23.07±1.27	5						
F+CT	5	23.18±1.23	3			2	Not run		
**Third Series: Birthday: 1-5 December in 2018**									
V+S	5	22.99±1.81	3	1	Unknown			1	illness
V+IT	5	22.85±2.41	5						
V+CT	5	23.15±0.48	5						
F+S	5	23.67±1.44	4	1	Unknown				
F+IT	5	23.71±1.41	3			2	Not run		
F+CT	6	23.76±1.09	6						
**Total**	79		63			16			

1. Data for body weight (BW) represent the mean ± S.D. There were no significant differences in BWs among the six groups in each series (one-way ANOVA with Tukey-Kramer method for multiple comparisons).
2. The causes of unexpected death or illness were not determined.
3. “Not run” was assigned to mice in both IT and CT groups that exhibited running avoidance behaviors more than three consecutive times, i.e., each ran less than ten minutes.

Disease Models & Mechanisms • Supplementary information

Table S2.

Click here to download Table S2

Table S3. Primers used for real-time qPCR.

Gene	Forward	Reverse
18S	GTAACCCGTTGAACCCCATTC	CCATCCAATCGGTAGTAGCG
IL6	ACAAAGCCAGAGTCCTTCAGAG	GCTTATCTGTTAGGAGAGCATTGG
Ppargc1a	AAGTGTGGAACTCTCTGGAACTG	GGGTTATCTTGGTTGGCTTTATG
Glut4	GGTCTCGGTGCTCTTAGTAGAACG	TGGCCACGATGGAGACATAGC
Pdk4	CACATGCTCTTCGAACTCTTCAAG	TGATTGTAAGGTCTTCTTTTCCCAAG
Apoa1	GTGGCTCTGGTCTTCCTGAC	ACGGTTGAACCCAGAGTGTC
Apoa2	GCCTGTTCACTCAATACTTTCAG	CAGACTAGTTCCTGCTGACC
Hspb1	TCACAGTGAAGACCAAGGAAGGC	GTGAAGCACCGAGAGATGTAGCC
Hspa1b	CAGCGAGGCTGACAAGAAGAA	GGAGATGACCTCCTGGCACT
Cryab	CTGCAGGCAGGACATAGGTG	CCGCCAGTTCATGGAGACTT
Gadd45g	ACTCTGGAAGAAGTCCGTGG	GGACTTTGGCGGACTCGTAG
Cdkn1a	GCTGTCTTGCACTCTGGTGTC	CAATCTGCGCTTGGAGTGATAG
G6pc	ATGGTCACTTCTACTCTTGC	CAAGATGACGTTCAAACAC
